# A comprehensive characterization of agronomic and end-use quality phenotypes across a quinoa world core collection

**DOI:** 10.3389/fpls.2023.1101547

**Published:** 2023-02-16

**Authors:** Evan B. Craine, Alathea Davies, Daniel Packer, Nathan D. Miller, Sandra M. Schmöckel, Edgar P. Spalding, Mark Tester, Kevin M. Murphy

**Affiliations:** ^1^ The Land Institute, Salina, KS, United States; ^2^ Department of Chemistry, University of Wyoming, Laramie, WY, United States; ^3^ Sustainable Seed Systems Laboratory, Department of Crop and Soil Sciences, Washington State University, Pullman, WA, United States; ^4^ Department of Botany, University of Wisconsin-Madison, Madison, WI, United States; ^5^ Department Physiology of Yield Stability, Institute of Crop Science, Faculty of Agriculture, University of Hohenheim, Stuttgart, Germany; ^6^ Division of Biological and Environmental Sciences and Engineering (BESE), King Abdullah University of Science and Technology (KAUST), Thuwal, Saudi Arabia

**Keywords:** quinoa, plant breeding, protein, amino acids, high-throughput phenotyping

## Abstract

Quinoa (*Chenopodium quinoa* Willd.), a pseudocereal with high protein quality originating from the Andean region of South America, has broad genetic variation and adaptability to diverse agroecological conditions, contributing to the potential to serve as a global keystone protein crop in a changing climate. However, the germplasm resources currently available to facilitate quinoa expansion worldwide are restricted to a small portion of quinoa’s total genetic diversity, in part because of day-length sensitivity and issues related to seed sovereignty. This study aimed to characterize phenotypic relationships and variation within a quinoa world core collection. The 360 accessions were planted in a randomized complete block design with four replicates in each of two greenhouses in Pullman, WA during the summer of 2018. Phenological stages, plant height, and inflorescence characteristics were recorded. Seed yield, composition, thousand seed weight, nutritional composition, shape, size, and color were measured using a high-throughput phenotyping pipeline. Considerable variation existed among the germplasm. Crude protein content ranged from 11.24% to 17.81% (fixed at 14% moisture). We found that protein content was negatively correlated with yield and positively correlated with total amino acid content and days to harvest. Mean essential amino acids values met adult daily requirements but not leucine and lysine infant requirements. Yield was positively correlated with thousand seed weight and seed area, and negatively correlated with ash content and days to harvest. The accessions clustered into four groups, with one-group representing useful accessions for long-day breeding programs. The results of this study establish a practical resource for plant breeders to leverage as they strategically develop germplasm in support of the global expansion of quinoa.

## 1 Introduction

Quinoa (*Chenopodium quinoa* Willd.), a pseudocereal developed and stewarded by indigenous communities of the Andean Region of South America for the past 7,000 years, is gaining worldwide attention for its potential to produce seed when grown under marginal conditions that can be flavorful and have numerous nutritional and health benefits (Dillehay et al., 2007; Bazile et al., 2016). Quinoa is an allotetraploid annual plant in the Amaranthaceae family, with a base chromosome number of *x* = 9 (2*n* = 4*x* = 36) (Jarvis et al., 2017). As a pseudocereal, quinoa is cultivated for the edible portions of the grains. The dispersal unit is the grain botanically referred to as an achene, which is a dry, one-seeded fruit that consists of a single seed enclosed in a dry and indehiscent pericarp (Burrieza et al., 2014). In quinoa, the pericarp is extremely thin, consistently of two single cell layers, and thus can be referred to as utricle (Prego et al., 1988). Quinoa protein quality can be superior to wheat, barley and soybean (Angeli et al., 2020). Quinoa is typically regarded as a complete protein, because it usually contains all nine of the essential amino acids in adequate proportions to meet human health requirements. However, quinoa has been shown to have limiting amino acid content, where amino acid content fails to meet daily requirements for leucine, lysine, tryptophan, aromatic amino acids, threonine, valine, and methionine (Mahoney et al., 1975; Ruales and Nair, 1992; Boye et al., 2012; Gonzalez et al., 2012; Craine & Murphy, 2020). Therefore, in these instances, quinoa cannot be considered a complete protein. A balance of fatty acids, minerals, vitamins, antioxidants and dietary fiber also contributes to the exceptional nutritional value of quinoa (Vega-Gálvez et al., 2010). Consumption is limited by the presence of saponins in the outer layers of the seed (e.g. 87% found in the pericarp), which must be removed before consuming, although saponin free or “sweet” varieties do exist (Filho et al., 2017; Jarvis et al., 2017; Zhao et al., 2022). Quinoa is tolerant to salinity and drought stress, in addition to other abiotic stressors, which are likely to become increasingly important under a changing climate (Hinojosa et al., 2018; Hajihashemi et al., 2022; Huan et al., 2022). Quinoa has the potential to be incorporated into a diverse range of end-uses, from traditional and non-traditional applications to industrial innovations, and improve functional properties and nutritional quality.

Quinoa germplasm is highly diverse. Considerable variability exists for physiological (e.g. drought and salinity tolerance, water use efficiencies, and photoperiod sensitives), morphological (e.g. various plant and panicle architectures, grain sizes and colors, growth habits), seed composition (e.g. protein content, protein composition) and end-use quality characteristics (Aluwi et al., 2017; Wu et al., 2017a; Wu et al., 2017b; Murphy et al., 2018). Domestication and selection of quinoa under variable conditions has contributed to natural variability and facilitated adaptation to diverse agroecological conditions. Independent domestication events gave rise to two germplasm pools, one in the Andean highlands and the other in the central and southern Chilean coastal lowlands (Maughan et al., 2019; Patiranage et al., 2022). While these areas represent major centers of diversity, the natural range of quinoa extends from southern Colombia (0°S) to southern Chile (40°S), and within this range, quinoa is cultivated from sea level to 4,000 meters above sea level (m.a.s.l) (Zurita-Silva et al., 2014). Quinoa is traditionally classified into five ecotypes based on geographic distribution; each ecotype is associated with defining characteristics (Tapia, 2015).

The broad genetic variability and adaptability of quinoa has produced a gene pool able to support strategic germplasm development. This effort, to develop quinoa varieties suitable for adoption in novel agroecological climates worldwide, is currently underway. Quinoa improvement has only recently benefited from the focus of formal breeding programs initiated within and outside of the Andes in the 1960s and 1970s, respectively (Zurita-Silva et al., 2014; Hinojosa et al., 2021). Recently, Jarvis et al. (2017) published a quinoa reference genome for a coastal Chilean quinoa accession (PI-614886; “QQ74”), providing the foundation from which to elucidate the genetic architecture of desirable traits and to support accelerated improvement through targeted breeding efforts (i.e. marker-assisted selection) (López-Marqués et al., 2020). While there are over 16,000 accessions of quinoa conserved across 30 countries within 50 germplasm banks, the vast majority of this germplasm is concentrated in South America and is not readily accessible outside of the region (Rojas et al., 2015; Bazile et al., 2016; Hinojosa et al.,2018). Quinoa is not included within the multilateral system of access and benefit sharing established by the International Treaty on Plant Genetic Resources for Food and Agriculture (ITPGRFA) and a clear legal framework for the equitable exchange of germplasm does not exist (Chevarria-Lazo et al., 2015). As evidenced by the collection dates of quinoa accessions within the USDA National Plant Germplasm System (NPGS), a significant portion of South American quinoa germplasm was collected and shared prior to the Convention on Biological Diversity in 1992 when national sovereignty over genetic resources was codified. Current access to South American germplasm must be negotiated with national governments and often requires facilitation by international organizations such as the Food and Agriculture Organization (FAO) of the United Nations (Maliro et al., 2021).

Despite these limitations, a quinoa “world core collection” has been developed by a team at the King Abdullah University of Science and Technology under the direction of Dr. Mark Tester. The collection, originally opportunistically assembled to capture representative geographic diversity as a proxy for genetic diversity, is available to researchers and breeders working to realize the potential of quinoa to respond to global challenges. Limited access to germplasm outside of South America is one factor that restricted the representativeness of the collection, relative to the overall diversity of quinoa. Target traits for adaptation will vary by region and will include agronomic traits such as abiotic and biotic stress resistance, plant height, days-to-maturity, and seed yield. End-use quality traits such as protein content and composition, seed size and shape, thousand seed weight (TSW), and seed color are also important (Murphy et al., 2018). There is potential to increase seed yield through indirect selection of certain traits (e.g. plant height, seed size, TSW) (Bertero and Ruiz, 2008; Bhargava et al., 2007a). This study aimed to *i*) characterize the “world core collection” for agronomic and end-use quality traits under long-day greenhouse conditions, *ii*) identify relationships between agronomic and end-use quality traits, and *iii*) calculate best linear unbiased estimators (BLUEs) and broad-sense heritability values to provide much needed information in support of quinoa research, development and production in non-traditional regions. Comparisons between variety trial (VT) entries, representing advanced breeding lines and commercial varieties from the Washington State University (WSU) Sustainable Seed Systems Laboratory (SSSL) 2018 Quinoa Variety Trial (*N* = 26), and accessions from the World Core Collection (WCC) (*N* = 334), are given special attention and extrapolated to consider the potential benefits to long-day breeding programs. Given the continued evaluation and selection of the WSU Quinoa Breeding Program, we expect these entries to be well adapted to long-day conditions and to provide a strong basis from which to make comparisons to a mostly novel set of germplasm.

## 2 Materials and methods

### 2.1 Germplasm, study design and greenhouse conditions

The world core collection of quinoa accessions (*N* = 334) and 26 entries in the 2018 WSU quinoa variety trial, were planted in a randomized complete block design with four replicates in each of two greenhouses on the WSU-Pullman campus. Additional information for the accessions is provided in **Supplementary Table S1**. Replicates were oriented in the greenhouse perpendicular to the direction of cooling (swamp cooler-exhaust fan axis). The greenhouses were planted approximately one month apart; greenhouse A (i.e. 7B) was planted on May 17th, 2018 and greenhouse B (i.e. 34B) was planted on June 14, 2018. Each greenhouse received 16 hours of light and 8 hours of dark, supplied through supplemental lighting, for the duration of the study. The temperature was maintained at 20°C during the day and 15°C at night, with relative humidity ranging from 40-70%. Three seeds of each accession were sown approximately 5mm below the surface of a soil-less media in each pot (2.6L). The pots were prepared with the same volume of media, which includes dolomitic limestone remnants and 70-80% Canadian Sphagnum peat moss (Sunshine Professional Growing, Agawam, MA, USA, www.agawam.ma.us). Plants were watered to saturation every other day.

Once two sets of true leaves were fully expanded on one plant in each pot, the remaining plants were removed by cutting the stem at the surface of the media. This resulted in a single plant per pot. Therefore, “plant” or “plants” refers to either a single replicate of one accession, or the collective replicates of accessions. This process was initiated approximately 14 days after sowing (DAS) for each greenhouse, and pots that did not have emerged plants were replanted. Each plant was fertilized with 2 g of Osmocote^®^ classic (13‐13‐13; Everris, NA). Even with a delay in sowing dates between the two greenhouses, fertilizer application occurred at approximately the same growth stage. For greenhouse A, fertilizer application occurred 28 days after sowing, while fertilizer application for greenhouse B occurred 27 days after sowing. Throughout the growing period, biological and chemical controls were applied to manage pest populations (all treatments listed in **Supplementary Table S2**). To mitigate the risk of stem breaking, plants were secured to a bamboo stake using a plastic-coated wire once they reached a height of approximately 90cm.

### 2.2 Greenhouse phenotyping

Two height measurements, from the top of the pot to the apex of the primary meristem, were recorded for each greenhouse during anthesis. The first and second measurements were taken at 36 and 43 DAS, respectively, for greenhouse 7B and at 35 and 41 DAS, respectively, for greenhouse 34B. Replanted samples were not included in analysis of these height measurements, because this measurement was made on two specific dates during flower and after sowing when all other plants had the same number of days of growth. They are omitted because we did not record height at 5 and 6 weeks after sowing. However, the replanted plants were included in all other analyses. Height at harvest was dependent on physiological maturity. Following the onset of anthesis in each greenhouse, all plants were evaluated every other day for the following growth stages: anthesis (i.e. BBCH 60), fruit set/ripening (i.e. BBCH 70), and physiological maturity (i.e. BBCH 89) (Sosa-Zuniga et al., 2017). At least one flower on the plant had to have fully extruded stigmas for 60 to be recorded; one ovary had to be fully ripened for 70 to be recorded; and one seed in the top third of the panicle had to be difficult to crush with a fingernail for 89 to be recorded. These methods agree with those proposed by Stanschewski et al. (2021) for BBCH 60. Once a plant reached stage BBCH 89, it was tagged with colored marking tape corresponding to the day, and all plants tagged on that day were harvested approximately two weeks later. This delay was intended to provide time for the remainder of seeds in the panicle to reach physiological maturity.

### 2.3 Harvest phenotyping

Several measurements were performed at harvest. The height of the plant, from the top of the pot to the apex of the primary meristem, was recorded. The panicle(s) was cut at the base and the length and width of the panicle were recorded. Digital images of each panicle were then captured inside of a light box (**Supplementary Figures S1A–C**). The light box was constructed from a cardboard box, two LED light strips, and a matte black background. A size and reflectance standard were positioned below the panicles. Up to three panicles were imaged simultaneously, although some panicles were imaged individually. Rarely was a panicle too large to be imaged effectively in the box. These images served as the basis from which to score inflorescence color (**Supplementary Figure S2**), shape, density, and leafiness, and provide a catalogue of reference images for each accession. The panicle phenotype scores used for shape, density, and leafiness are detailed by Stanschewski et al. (2021), where the phenotyping cards are provided in the supplementary material.

### 2.4 Postharvest phenotyping

Each panicle was placed in a labeled paper bag after photographing. Panicles were allowed to dry on metal greenhouse benches for 2-3 weeks before being threshed individually by hand using latex-coated gloves. Metal screens were used to sieve the threshed material to remove non-seed material and to gently abrade the seed to remove the pericarp and any adhering opercula. A Holland BV seed blower type 4110.21.00 (200 mm) with inlet cup of 125 mm model 4110.20.09 (Seed Processing Holland B.V. Enkhuizen, The Netherlands) was used to remove fine debris and produce a sample for each plant consisting solely of seed. Seed is used here and hereafter to refer to the physical material produced from this process as described. Given the diverse germplasm used in this study, this process resulted in seeds that could have had intact pericarp or integument. While the degree of clean seed was not quantified and appeared to vary to a small degree both within and among accessions, the clean seed can be seen in **Supplementary Figure S3**. These whole, unprocessed (i.e. no additional abrasion or washing) seed samples from each plant were analyzed to predict crude protein, crude fat, ash, total carbohydrate and moisture content, in addition to a complete amino acid profile, using a PerkinElmer (formerly Perten) DA7250 Near-Infrared Spectrometer with a near-infrared (NIR) range of 950-1650 nm and absorbance values recorded at every 5 nm (PerkinElmer, Waltham, MA, USA). Development and validation of the instrument calibration is described in Stanschewski et al. (2021). Official methods of analysis and analytical data for 100 out of the 175 reference samples used to develop the calibration are provided in Craine and Murphy (2020). These reference samples (*N* = 175) are not included in this study and represent an external data set from which seed components are predicted for the novel samples included in this study. Samples with predicted values outside of the respective ranges provided in **Table 1** for moisture, ash, crude protein, crude fat, total amino acids, and in **Table 2** for each amino acid were first filtered to exclude these samples. The second filtering step consisted of removing spectra (representing experimental samples) that had a Mahalanobis distance significantly different from the calibration (i.e. reference) spectra (*p<* 0.001). *P* values were calculated using alpha equal to 0.001 and a χ^2^ distribution with degrees of freedom equal to the total number of measured wavelengths in the NIR range (*n* = 141). The covariance matrix was calculated using the raw spectra values from both the experimental and calibration samples, while the centroid represented mean absorbance values (raw spectra) at each wavelength in the NIR range for the calibration samples. These filtering steps were used to identify outliers that were then excluded from the analysis of seed composition data predicted *via* NIR.

**Table 1:** Washington State University (WSU) Sustainable Seed Systems Laboratory (SSSL) NIR calibration (V3) metrics for primary seed components.

Primary Seed Components^a^	Range	Min	Max	RMSECV^b^	SECV^b^	Robust SECV^b^	RPDCV^b^	R^2^CV^b^
Crude Protein	11.95	7.558	20.658	0.394	0.395	0.406	5.521	0.967
Ash	3.32	2.449	6.156	0.154	0.154	0.129	3.084	0.895
Crude Fat	6.95	0.000	7.702	0.31	0.311	0.316	3.883	0.934
Crude Fiber	13.67	1.593	21.133	0.442	0.443	0.377	4.904	0.958
Moisture	3.76	6.410	10.170	0.183	0.183	0.159	6.579	0.977
Total AA	10.06	6.235	17.499	0.413	0.413	0.328	4.018	0.938

a Primary seed components reported as g 100g^-1^ sample dry matter content.

c RMSECV, root mean square error of cross validation; SECV, standard error of cross validation; RPDCV, ratio of reference data standard deviation to standard error of prediction; R^2^CV, coefficient of determination of cross validation. The range, minimum (min) and maximum (max) are calculated using reference data for quinoa samples included in the calibration (*N* = 175) (Craine and Murphy, 2020 and unpublished data). Calibration prediction accuracy metrics are reported as an average measure of 8-fold cross validation in triplicate. Calibration development is detailed in Stanschewski et al. (2021).

**Table 2:** Washington State University (WSU) Sustainable Seed Systems Laboratory (SSSL) NIR calibration (V3) metrics for each amino acid.

Amino Acids^a^	Range	Min	Max	RMSE CV^c^	SE CV^c^	Robust SE CV^c^	RPD CV^c^	R^2^CV^c^
Alanine	0.483	0.310	0.792	0.022	0.022	0.018	3.036	0.892
Arginine	4.68	0.475	1.849	0.053	0.053	0.044	4.308	0.946
Aspartic acid	3.22	0.586	1.618	0.039	0.04	0.036	3.768	0.93
Cysteine	0.76	0.111	0.341	0.01	0.01	0.01	3.188	0.902
Glutamic acid	7.04	0.818	2.961	0.093	0.093	0.086	3.802	0.931
Glycine	1.33	0.420	1.123	0.041	0.041	0.036	2.447	0.834
Histidine	1.05	0.177	0.594	0.015	0.015	0.014	4.564	0.952
Isoleucine	1.51	0.299	0.792	0.021	0.022	0.019	3.392	0.913
Leucine	2.55	0.453	1.222	0.031	0.032	0.029	3.473	0.917
Lysine	3.14	0.409	1.079	0.029	0.029	0.033	3.29	0.908
Methionine	1.15	0.133	0.374	0.012	0.012	0.009	2.955	0.886
Phenylalanine	1.57	0.287	0.781	0.019	0.019	0.018	3.889	0.934
Proline	1.68	0.265	0.704	0.023	0.023	0.018	2.556	0.847
Serine	1.39	0.265	0.726	0.019	0.019	0.016	3.176	0.901
Taurine	1.96	0.128	0.242	0.012	0.012	0.009	1.669	0.645
Threonine	1.6	0.254	0.638	0.017	0.017	0.016	3.015	0.89
Tryptophan	0.93	0.067	0.186	0.012	0.012	0.009	1.681	0.647
Tyrosine	0.93	0.221	0.539	0.014	0.014	0.013	3.393	0.913
Valine	1.84	0.354	0.902	0.024	0.024	0.023	3.26	0.906
Hydroxy lysine^b^	0.18	0.000	0.033	0.004	0.004	0.003	1.591	0.605
Hydroxy proline^b^	0.93	0.032	0.145	0.01	0.01	0.011	1.821	0.699

a Amino Acid (AA) values reported as g 100g^-1^ protein.

b Hydroxylysine and Hydroxyproline are poorly predicted and not included in analyses.

c RMSECV, root mean square error of cross validation; SECV, standard error of cross validation; RPDCV, ratio of reference data standard deviation to standard error of prediction; R^2^CV, coefficient of determination of cross validation. The range, minimum (min) and maximum (max) are calculated using reference data for quinoa samples included in the calibration (*N* = 175) (Craine and Murphy, 2020 and unpublished data). Calibration prediction accuracy metrics are reported as an average measure of 8-fold cross validation in triplicate. Calibration development is detailed in Stanschewski et al. (2021).

To record seed yield (hereafter yield), a sample of cleaned seed from each plant (representing a single replicate) was weighed to the nearest mg. A subsample of 1-2 grams was removed and weighed. This subsample was then scattered on a flatbed scanner and an 8-bit red, green, blue (RGB) image was captured at a resolution of 1,200 dots per inch (dpi). These images were then analyzed in the Cyverse Discovery Environment (http://de.cyverse.org/de) using the All Grains tool from the phytoMorph Image Phenomics Toolkit. The All Grains tool counted the individual seeds represented in the image, including those touching each other in clusters, using the approachdeveloped for counting maize kernels in similar images (Miller et al., 2017). The tool also returned the average seed area, major axis (length), minor axis (width), and eccentricity (length:width ratio) using the approach developed for Arabidopsis seeds by Moore et al. (2013). The tool measured the red, green, and blue (i.e. RGB) intensity values of each pixel within each seed and returned the sample average. The average RGB values were then multiplied by 255 to generate the corresponding RGB decimal code, which was used to quantitatively determine seed color within the RGB color model. Furthermore, the RGB values were added together to determine the total RGB value (i.e. sumRGB). TSW was determined by dividing the measured sample mass by the algorithmically-counted seed number, then multiplying this value by 1,000. This computed TSW strongly correlated with hand-counted values (**Supplementary Figure S3**). Total seeds per plant were calculated by dividing yield per plant by the weight of one thousand seeds. Protein yield was determined by multiplying crude protein content (g 100g sample^-1^ fixed at 14%) by seed yield (g plant^-1^).

### 2.5 Statistical analyses

All statistical analyses were performed using the R statistical software, unless otherwise noted (R Core Team, 2021). Incomplete or missing data were omitted from analyses. All data points, even values of 0.000 g, were included in yield analyses. Samples with a yield per plant value less than 0.5 g were excluded only from seed composition analyses (*N* = 95), while samples with less than 97 seeds in the seed images were excluded only from seed morphology analyses (*N* = 31). Certain seed images failed image analysis due various algorithm errors and were not included in the seed morphology data set (*N* = 21). Moreover, image analysis errors were identified *via* visual inspection of quality control images, and seed morphology data belonging to samples with errors were not included (*N* = 23). Replanted samples were not included in analysis of height measurements, because this measurement was made on two specific dates during flower and after sowing when all other plants had the same number of days of growth. They are omitted because we did not record height at 5 and 6 weeks after sowing. However, the replanted plants were included in all other analyses. Height at harvest was dependent on physiological maturity. Pearson’s and Spearman’s correlation coefficients were calculated using the *rcorr* function in the *Hmisc* package (Frank and Dupont, 2021). A heatmap representing the correlation matrix was generated using the heatmap function form the stats package.

The following linear mixed model was used *y_ijk_* = *μ* + *α_i_* + *τ_j_* + (*ατ*)*_ij_* + *γ_jk_* + *ε_ijk_*, where y_ijk_ is the response variable observed in the k^th^ block (i.e. replicate) of the i^th^ genotype in the j^th^ environment (i.e. greenhouse); *μ* is the grand mean; *α_i_* is the effect of the i^th^ genotype; *τ_j_* is the effect of the j_th_ environment; (*ατ*)_*ij*_ is the interaction effect of the i^th^ genotype with the j^th^ environment; *γ_jk_* is the effect of the k^th^ block within the j^th^ environment; and ε_ijk_ is the random error. All factors were treated as random effects to estimate variance components and to calculate Cullis heritability according to Schmidt et al. (2019) using the best linear unbiased predictors (BLUPs), while accessions was treated as a fixed effect to estimate best linear unbiased estimates (BLUEs). Furthermore, standard heritability was calculated according to Schmidt et al. (2019) using the equation:


$$H_{standard}^{2}=\frac{\sigma_{g}^{2}}{\sigma_{p}^{2}};\;\sigma_{p}^{2}=\sigma_{g}^{2}+\frac{\sigma_{env}^{2}}{n_{env}}+\frac{\sigma_{g\;x\;env}^{2}}{n_{env}}+\frac{\sigma_{rep}^{2}}{n_{rep}}+\frac{\sigma_{err}^{2}}{n_{gen}*\;n_{env}*n_{rep}}$$

where $H_{standard}^{2}$ is the standard heritability, and $\sigma_{g}^{2}$ , $\sigma_{env}^{2}$ , $\sigma_{g\;x\;env}^{2}$ , $\sigma_{rep}^{2}$ , and $\sigma_{err}^{2}$ are the variance components of the genotype (i.e. accession) main effect, the environment (i.e. site year) main effect, the genotype by environment interaction effect, the replicate within environment main effect, and the error, respectively, and *n*_*gen*_ , *n*_*env*_ , *n*_*rep*_ is the number of genotypes, environments, and replicates within environments. Amino acid scores (AAS) were calculated by dividing the amino acid value (adjusted to 14% moisture; mg g^-1^ protein) by the respective daily requirement for the target age group (FAO/WHO/UNU, 2007).

Using a subset of the traits studied (days to anthesis, fruit set/ripening, days to harvest, height at 5 weeks and 6 weeks after sowing, height at harvest, inflorescence length, width and area, ash, crude fat, crude protein, total amino acids, yield per plant, TSW, seed area, eccentricity, and total RGB), principal component analysis was performed using prcmomp function in the stats package with data (i.e. BLUEs) centered and scaled, and principal component data were visualized graphically using the fviz_pca function in the factoextra package (Kassambara and Mundt, 2020). Following principal component analysis, agglomerative hierarchical cluster analysis was performed using BLUEs of accessions by first calculating a Euclidean distance matrix, with Ward’s method for clustering carried out using hclust. For the cluster analysis performed using yield, days to harvest, and height at harvest, the kmeans function from the stats package was used (R Core Team, 2021).

## 3 Results and discussion

### 3.1 Descriptive statistics

Descriptive statistics for the greenhouse, harvest and postharvest traits are provided in **Table 3**. Considerable phenotypic variation among the accessions contributed to the ranges and standard deviations shown in **Table 3**. Of the phenological growth stages, days to harvest had the largest range (115 days), followed by fruit set and ripening (98 days) and days to anthesis (60 days). Height at harvest had a range of 285 cm, due in part to the presence of a few abnormally stunted replicates of the accessions (hereafter plants). Most plants had a height at harvest between 112 and 175 cm (one standard deviation of the mean) (**Supplementary Table S4**).

**Table 3:** Summary statistics provided for the traits studied, including the total number of data points (*N*), standard deviation (SD), minimum (min), and maximum (max).

Traits	*N*	Mean ± SD	Min	Max
Days to Anthesis	2568	51 ± 9	33	93
Anthesis (days)	2537	16 ± 6	2	60
FruitSet/Ripening (days)	2749	28 ± 14	4	102
Days to Harvest	2880	110 ± 23	72	187
Plant Height-5wk After Sowing (cm)	2854	43 ± 7	4	65
Plant Height-6wk After Sowing (cm)	2854	61 ± 10	8	96
Plant Height-Harvest (cm)	2879	144 ± 31	13	298
Inflorescence Length (cm)	2876	34 ± 12	7	81
Inflorescence Width (cm)	2876	10 ± 5	1	46
Inflorescence Area (cm)	2876	348 ± 218	23	2440
Ash Content (%)	2634	2.69 ± 0.26	2.08	4.19
Crude Fat Content (%)	2621	3.62 ± 0.72	0.0.5	6.61
Crude Protein Content (%)	2641	14.02 ± 1.69	8.46	28.26
Total Amino Acid Content (mg g^-1^ protein)^ψ^	2635	828.71 ± 20.74	735.1	936.45
Histidine Content (mg g^-1^ protein)^ψ^	2618	27.28 ± 0.73	24.29	30.7
Isoleucine Content (mg g^-1^ protein)^ψ^	2567	38.83 ± 1.19	33.62	43.43
Leucine Content (mg g^-1^ protein)^ψ^	2635	59.36 ± 2.39	38.6	68.73
Lysine Content (mg g^-1^ protein)^ψ^	2533	55.39 ± 2.87	46.59	65.06
SAA Content (mg g^-1^ protein)^ψ^	2360	36.76 ± 1.47	28.55	41.88
AAA Content (mg g^-1^ protein)^ψ^	2575	63.8 ± 2	47.43	70.68
Threonine Content (mg g^-1^ protein)^ψ^	2620	31.38 ± 1.48	26.92	36.2
Tryptophan Content (mg g^-1^ protein)^ψ^	2346	9.96 ± 0.6	7.87	12.22
Valine Content (mg g^-1^ protein)^ψ^	2578	44.4 ± 1.81	37.52	51.28
Crude Protein Yield (g plant^-1^)	2640	0.8 ± 0.33	0.07	2.16
Yield (g plant^-1^)	2880	5.516 ± 2.821	0	14.18
Thousand Seed Weight (g)	2803	2.604 ± 0.687	0.816	4.739
Total Seeds (seeds plant^-1^)	2803	2170 ± 969	0	6358
Seed Area (mm^2^)	2804	2.54 ± 0.49	1.16	3.92
Seed Eccentricity	2804	0.35 ± 0.03	0.25	0.49
Total Red, Green, Blue	2804	377 ± 53	118	479

ψ, fixed at 14% moisture content.

At harvest, most plants had reached physiological maturity, allowing for relevant panicle phenotyping. Stanschewski et al. (2021) recommend harvesting when the entire panicle is at stage BBCH 89. However, they note that this stage can be difficult to score, especially when observing day-length sensitive plants that can exhibit regrowth in the panicle (i.e. stay-green trait). All possible inflorescence colors were observed, except for brown, black, red and white, and red and pink (**Supplementary Figure S2**). Most plants had green panicles (67%), followed by yellow (12%) and beige (7%) (data not shown). Inflorescence color (i.e. plant color) is a dominant morphological marker, and is a useful qualitative trait for confirming the successful production of F1 plants from crosses (Peterson et al., 2015). Most plants had an intermediate panicle shape (77%), although glomerulate (15%) and amarantiform (8%) panicles were observed (data not shown). An extremely small number of plants had dense (7) panicles (2%) (**Supplementary Figure S2**). Most plants had lax (1) (28%), intermediate (3) (34%) or intermediate (5) (36%) panicle density. Approximately half of the plants had leaves present in the panicles, scored as 3 (31%), 5 (23%) and 7 (2%), while 44% of plants had minimal to no leaves present (scored as 1). Manjarres-Hernández et al. (2021) performed panicle phenotyping for 30 accessions of quinoa, under greenhouse conditions with as 12:12 photoperiod, which belonged to the seed collection of the Department of Boyacá, Colombia. At physiological maturity, they observed panicle colors of purple, pink, yellow, orange, red, green, and a mixture between those colors. The majority of the plants (93%) had glomerulate panicle shape, compared to intermediate and amarantiform (7%). The difference in our results could be due to differences in classification for each category since it is a qualitative and subjective assessment. They also found a small percentage of dense panicles (10%), followed by intermediate (29%) and lax (61%).

### 3.2 Principal component analysis and clustering

**Figure 1:**
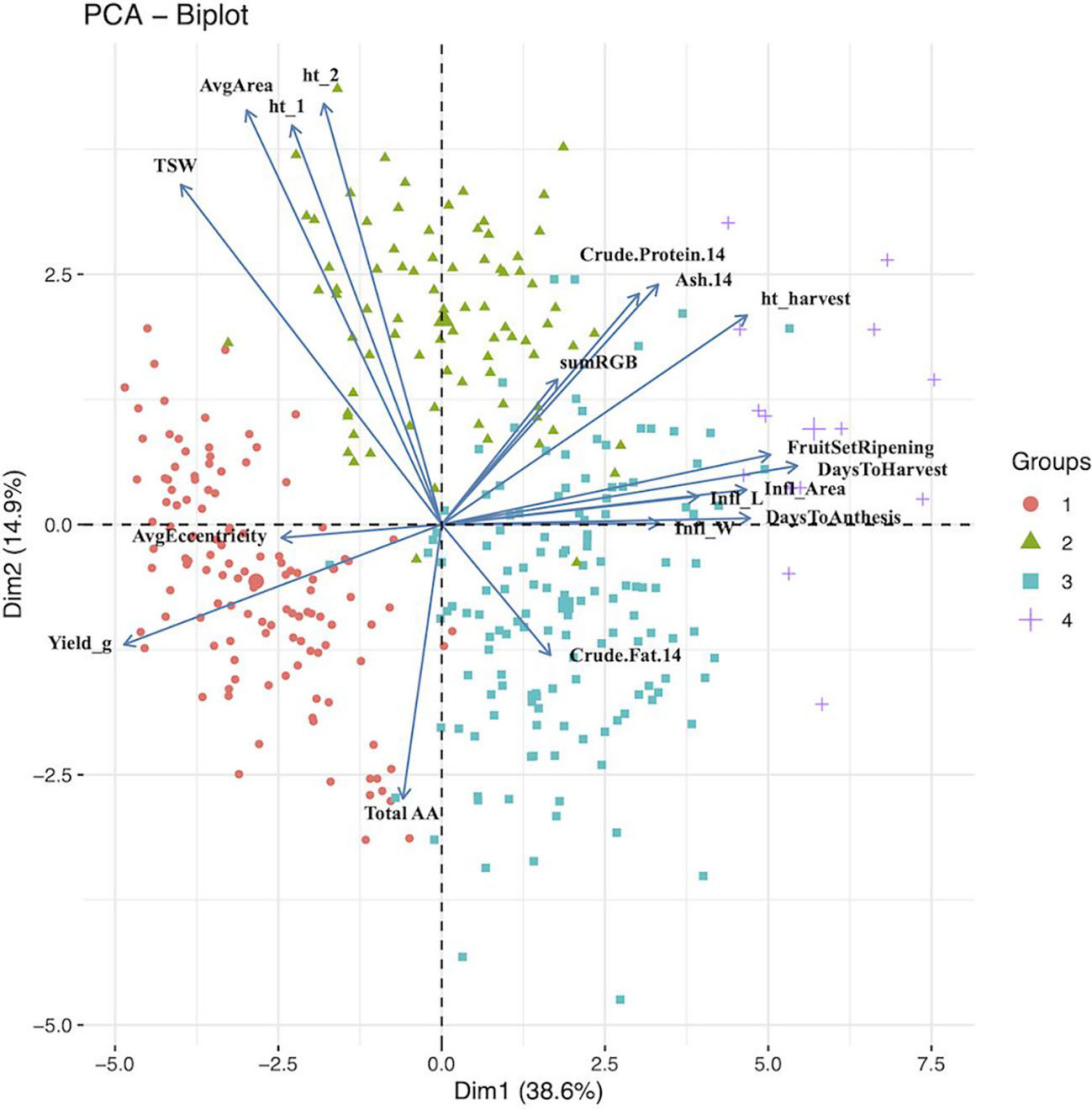
Principal component analysis biplot for dimensions 1 (Dim1) and 2 (Dim2). The groups (n =4) are color and shape coded according to agglomerative cluster analysis using Ward’s method. The larger shape for each group represent the group centroid. 14, 14% moisture; Infl, inflorescence; ht_1, height 5 weeks after sowing; ht_2, height 6 weeks after sowing; TSW, thousand seed weight; Avg, average; AA, amino acids.

**Figure 2:**
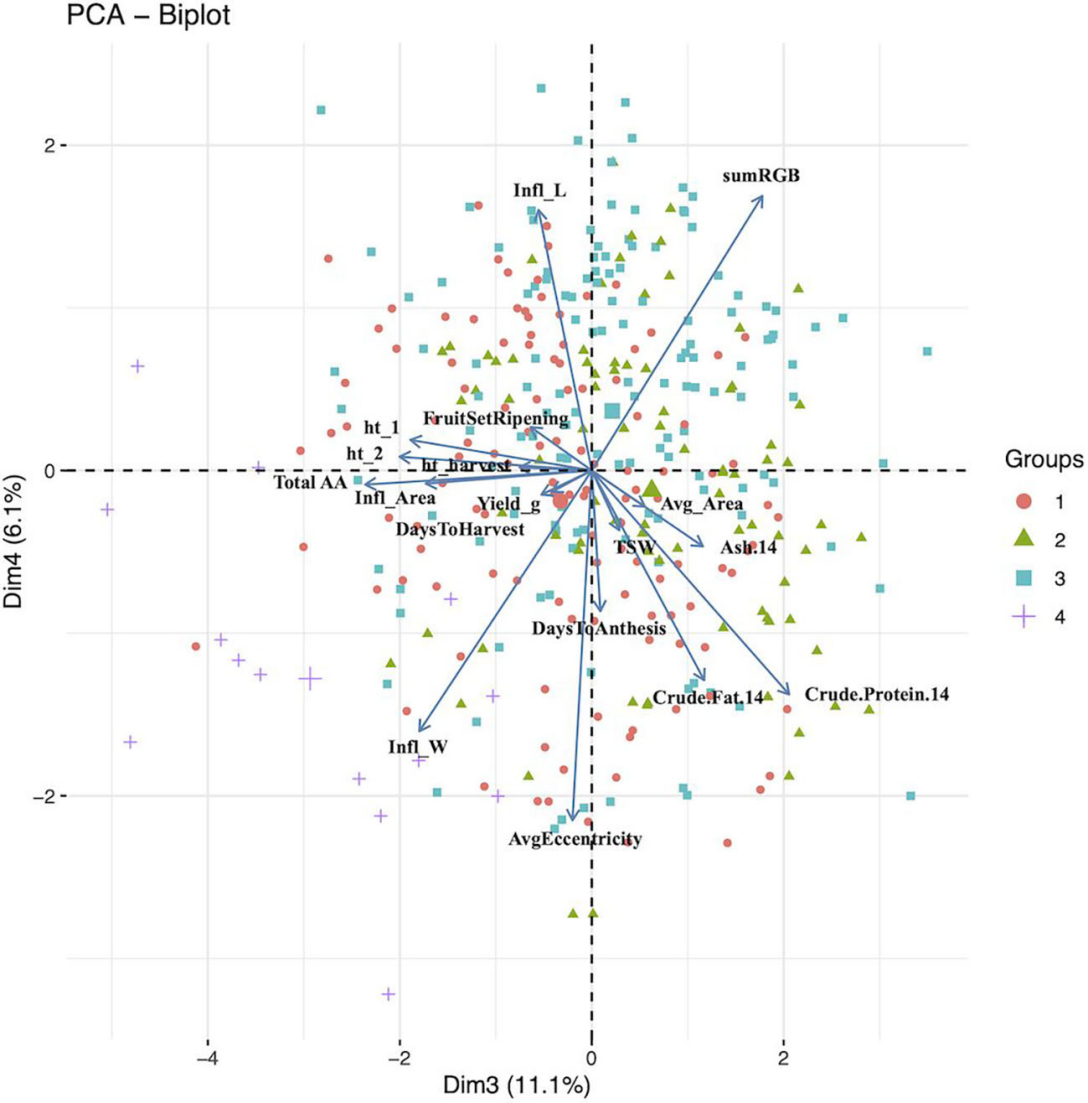
Principal component analysis biplot for dimensions 3 (Dim1) and 4 (Dim2). The groups (n =4) are color and shape coded according to agglomerative cluster analysis using Ward’s method. The larger shape for each group represent the group centroid. 14, 14% moisture; Infl, inflorescence; ht_1, height 5 weeks after sowing; ht_2, height 6 weeks after sowing; TSW, thousand seed weight; Avg, average; AA, amino acids.

Principal component analysis (PCA) provided a better understanding of how the traits contributed to the overall variance observed and possible ways to characterize the germplasm using these traits. The traits included in the PCA are shown in **Table 4** along with their loading values. Moreover, results from the first five principal components are provided in **Table 4**. The corresponding scree plot is show in **Supplementary Figure S5**, and PCA biplots for PC1 and PC2, and PC2 and 3 in **Figure 1** and **Figure 2**, respectively. The plane formed by the first two dimensions explains 53.45% of the total variability for the cloud of trait data (i.e. 53.45% of the total dataset inertia). For each dimension, variables that had contributions greater than the threshold value (5.56%; expected value if the contributions were uniform across variables) are reported in decreasing order according to percent contribution. Days to harvest, yield/plant, fruit set/ripening, days to anthesis, height at harvest, inflorescence area, TSW, inflorescence length, and total amino acid content had greater contributions to dimension one (**Table 4**). Bhargava et al. (2007b) had similar findings, with the greatest dimension one loading values belonging to inflorescence/plant, plant height, leaf size and seed yield/plant.

**Table 4:** Principal component analysis (PCA) results, including loadings values for principal components (PC) one through five for each trait studied.

Traits	PC1 (38.56%)	PC2 (14.89%)	PC3 (11.07%)	PC4 (6.12%)	PC5 (5.54%)
Days to Harvest	0.354	0.061	-0.074	-0.031	0.013
FruitSet/Ripening	0.327	0.073	-0.113	0.064	-0.011
Yield	-0.316	-0.125	-0.093	-0.036	0.271
Days to Anthesis	0.307	0.007	0.016	-0.207	0.185
Plant Height - Harvest	0.304	0.218	-0.134	0.005	0.050
Inflorescence Area	0.303	0.036	-0.308	-0.019	-0.050
Thousand Seed Weight	-0.259	0.355	0.051	-0.088	0.086
Inflorescence Length	0.255	0.029	-0.099	0.383	-0.211
Inflorescence Width	0.216	0.001	-0.319	-0.383	0.132
Ash Content	0.215	0.251	0.206	-0.112	-0.188
Crude Protein Content	0.196	0.241	0.366	-0.329	-0.006
Seed Area	-0.194	0.433	0.098	-0.053	0.119
Seed Eccentricity	-0.160	-0.014	-0.036	-0.514	-0.546
Plant Height-6wk After Sowing	-0.149	0.417	-0.356	0.020	0.082
Plant Height-5wk After Sowing	-0.117	0.440	-0.336	0.045	0.137
Total Red, Green, Blue	0.115	0.152	0.316	0.404	0.186
Crude Fat	0.108	-0.137	0.208	-0.308	0.617
Total Amino Acid Content	-0.038	-0.286	-0.420	-0.021	0.172

The explained variance for each principal component (PC) is reported in parentheses in the column header. Rows are sorted by PC1 value, from largest to smallest absolute values for the traits studied.

For dimension two, the variables height at 5 weeks and 6 weeks after sowing, seed area, TSW, ash and total amino acid had contributions above the threshold (**Figure 1**). These results agree with those reported by Bhargava et al. (2007b), where seed morphological traits, such as TSW and seed size, and days to maturity had the largest coefficients. Total amino acid content, inflorescence area, ash content, inflorescence width, crude protein content, and height at 5 and 6 six weeks after sowing had contributions to dimension three above the threshold, with dimension four contributions above the threshold came from seed shape (i.e. eccentricity), crude fat content, anthesis, inflorescence length and days to anthesis. Finally, crude fat content, seed shape, crude protein content and anthesis had contributions to dimension five above the threshold (**Figure 2**).

**Figure 3:**
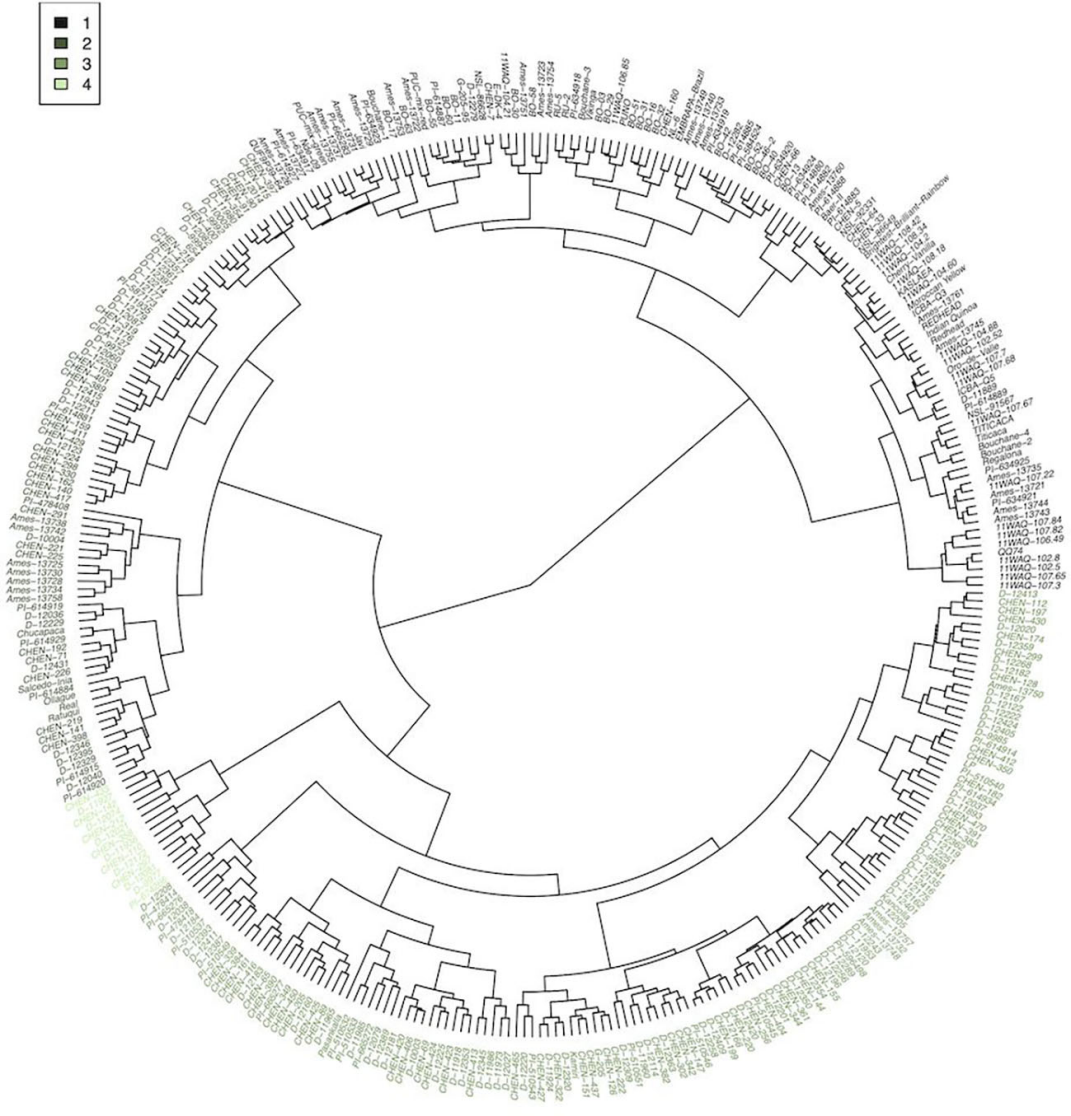
Agglomerative cluster analysis, calculated first with a Euclidean distance matrix and then Ward’s method for clustering, of accession best linear unbiased estimates using a subset of the phenotypic traits (days to anthesis, fruit set/ripening, days to harvest, height at 5 weeks and 6 weeks after sowing, height at harvest, inflorescence length, width and area, ash, crude fat, crude protein, total amino acids, yield per plant, TSW, seed area, eccentricity, and total RGB). The dendrogram was cut to form 4 groups, and each cluster (*N* = 4) is color coded according to the legend. Cluster identities are also provided in **Supplementary Table S4** under column ATC (i.e. all trait cluster).

Cluster analysis provided an additional approach to characterize and group the germplasm. Four groups were constructed, according to accession BLUEs for the traits included in the principal component analysis (**Figure 3**). Using a similar statistical approach, and 117 accessions grown in Faisalabad, Pakistan, Hafeez et al. (2022) also found four groups and report average phenotypic values by group. In our study, group 1, 2, 3, and 4 consisted of 122, 82, 139, and 14 accessions, respectively. Three accessions could not be assigned to a group because of missing data. Mean values for the traits varied among the groups (**Table 5**). Passport data, when available for the accessions, provides an estimate of the latitude of origin. Group 1, 2, 3, and 4 had a median absolute latitude of 46° (*N* = 37), 14° (*N* = 36), 16° (*N* = 68), and 13° (*N* = 6), respectively. The values tended to reflect possible germplasm adaptation to day-length, with average days to harvest of 91, 110, 121, and 159 for group 1, 2, 3, and 4, respectively. Hafeez et al. (2022) also found one group to have a shorter “cycle” (i.e. days to physiological maturity). In addition to having more days to harvest, group 4 had the greater height at harvest and panicle size. Groups 1 and 2 had similar TSW, with mean values greater than groups 3 and 4. Group 2 had larger seed size, although similar to group 1, with seed color similar to group 3 and closer to white than the other groups. Moreover, Group 2 had higher protein content compared to the other groups. Group 2 may represent varieties bred for the Altiplano region of Bolivia and Peru. It is possible that these Real-type quinoas have been selected to have large, white seeds according to prevailing domestic and export market standards (Fuentes et al., 2009). Chucapaca, Ratuqui, and Real represent notable commercial varieties in this group. For example, a draft genome has been published for Real (Zou et al., 2017). The former El Instituto Boliviano de Tecnología Agropecuaria (IBTA) in Bolivia (1967-1996) released Ratuqui in 1993 and Chucapaca in 1986 (FAO, 2011; Bonifacio, 2019). We observed seed area BLUE values of 2.57 mm^2^ for Chucapaca, 3.14 mm^2^ for Ratuqui, 3.39 mm^2^ for Real, compared to an average value of 2.53 mm^2^ (**Supplementary Table S4**). Fuentes et al. (2009) found that cluster analysis separated the central Andes accessions (i.e. Highland) from the southern latitude accessions (i.e. Coastal). In their study, the coastal group included three European varieties, likely originating from the southern Chilean coastal zone (Fuentes et al., 2009). Group 1 included all of the WSU VT entries, in addition to the WCC accessions that clustered with them. This group appears to be higher yielding, although with lower crude protein and ash content, and includes accessions that may be most relevant to long-day breeding programs given the more rapid maturation observed under the study conditions and the higher median absolute latitude. Several commercial varieties bred by Frank Morton of Wild Garden Seeds (https://www.wildgardenseed.com/index.php?cPath=50 ), a private breeding company located in Philomath, OR, USA adjacent to the WSU program’s target environments, are also included in group 1. This group also includes the varieties Puno, Titicaca, and Vikinga, which have been bred specifically for northern European environments by the company Quinoa Quality ApS (Regstrup, Denmark) (Präger et al., 2018), it is likely that the accessions comprising group 1 have parental origins in the southern Chilean coastal zone. For comparison, Thiam et al. (2021) found four groups through average linkage cluster analysis, with Vikinga, Titicaca, and Puno in one group.

**Table 5:** Mean values plus and minus (±) standard deviation reported for each trait within each of the clusters (N =4).

Traits	1 (N = 122)	2 (N = 82)	3 (N = 139)	4 (N = 14)
Days to Anthesis	46 ± 4	51 ± 5	54 ± 5	67 ± 9
Anthesis (days)	14 ± 2	17 ± 3	17 ± 3	16 ± 3
FruitSet/Ripening (days)	18 ± 2	28 ± 7	35 ± 9	52 ± 11
Days to Harvest	91 ± 5	110 ± 10	121 ± 14	159 ± 13
Plant Height-5wk After Sowing (cm)	44 ± 4	46 ± 3	40 ± 4	45 ± 3
Plant Height-6wk After Sowing (cm)	63 ± 6	65 ± 5	57 ± 5	64 ± 4
Plant Height - Harvest (cm)	117 ± 17	155 ± 17	154 ± 18	197 ± 22
Inflorescence Length (cm)	25 ± 4	35 ± 7	41 ± 7	42 ± 10
Inflorescence Width (cm)	9 ± 3	9 ± 2	10 ± 2	21 ± 3
Inflorescence Area (cm)	215 ± 69	325 ± 105	422 ± 104	873 ± 201
Ash Content (%)	2.54 ± 0.12	2.83 ± 0.16	2.74 ± 0.18	2.82 ± 0.11
Crude Fat Content (%)	3.53 ± 0.47	3.55 ± 0.49	3.73 ± 0.6	3.61 ± 0.61
Crude Protein Content (%)	12.96 ± 1.08	15.2 ± 1.08	14.22 ± 0.94	14.89 ± 1.04
Total Amino Acid Content (mg g-1 protein)^ψ^	836.2 ± 10.53	817.34 ± 14.34	828.14 ± 14.45	839.41 ± 17.66
Yield (g)	8.289 ± 1.133	4.387 ± 1.356	4.138 ± 1.228	2.182 ± 1.087
1,000 Seed Weight (g)	2.937 ± 0.5	3.022 ± 0.382	2.126 ± 0.313	2.056 ± 0.468
Seed Area (mm^2^)	2.687 ± 0.354	2.922 ± 0.281	2.207 ± 0.274	2.297 ± 0.327
Seed Eccentricity	0.356 ± 0.02	0.348 ± 0.03	0.336 ± 0.027	0.332 ± 0.021
Total Red, Green, Blue	356 ± 34	395 ± 32	384 ± 64	368 ± 37

Groups were identified using agglomerative hierarchical clustering with Ward’s method. The resulting dendrogram was cut to produce the four groups.

### 3.3 Days to harvest and inflorescence phenotypes

Northern European and North American breeding programs, such as the WSU program, generally develop germplasm for long-day environments. Reducing time to maturity, through introduction of adapted germplasm and careful selection of progeny, can help these programs overcome day-length sensitivity issues that exist within quinoa germplasm (Bazile et al., 2016). The WSU program aims to develop germplasm that produce mature seed in 90-100 days and all WSU variety trial (VT) accessions met this goal. However, in addition to producing mature seed in 90-100 days, the WSU programs aims for harvestability, which is a function of mature seed and fully senesced vegetative tissues, to occur at 120 days or less. A stay-green trait in quinoa may contribute to a greater extent of grain filling, while delaying days to harvest, and is possibly linked to photoperiod sensitivity (Christiansen et al., 2010).

Within the WSU VT entries, days to harvest ranged from a minimum of 84 days (Titicaca [WSU VT seed source]; sd = 7) to a maximum of 91 days (breeding line 11WAQ-108.42; sd = 9). An average of 88 days to harvest (sd = 8) for the WSU VT entires demonstrated ongoing efforts to select for early maturity in breeding lines and varieties. Comparatively, the World Core Collection (WCC) had an average of 112 days to harvest (min = 84 days; maximum = 177 days). However, 34 out of the 334 accessions had an average days to harvest less than the 90 day target average days to harvest within this range (data not shown). These included, in order of earlier to later, Moroccan Yellow, Titicaca (WCC seed source), BO-03, Bouchane-2, Bouchane-3, Ames-13743, Vikinga, EMBRAPA-Brazil, RU-2, E-DK-4, Cherry Vanilla, PI-614889, PI-634923, D-11889, BO-32, BO-63, NL-6, RU-5, Bouchane-1, Regalona, Brightest-Brilliant-Rainbow, ICBA-Q5, Oro-de-Valle, BO-29, PI-665276, PI-614927, BO-30, Bouchane-4, Ames-13722, and PI-634921. Three of these accessions are varieties released by Wild Garden Seeds (Cherry Vanilla, Brightest-Brilliant-Rainbow, and Oro-de-Valle) and two are varieties released by Quinoa Quality (Titicaca and Vikinga).

In addition to prolonged days to harvest, WCC accessions that likely suffered from day-length sensitivity exhibited a distinctive phenotype. These accessions generally had large, lax panicles with reduced flowering structures and consequently lower yield. For example, D-11927 had an average days to harvest of 169, an average inflorescence area of 972 cm^2^, and a median value for inflorescence leafiness of 5 (out of 7) (**Figure S6A**). This is compared to some of the earliest harvested plants, represented by Titicaca (VT & WCC seed source) and Bouchane-2 (**Figure S6B**). Both accessions for Titicaca had an average of 84 days to harvest, while Bouchane-2 had an average of 85 days. Titicaca had an average inflorescence area of 149 cm^2^ (VT seed source) and 148 cm^2^ (WCC seed source), while Bouchane-2 had an average of 122 cm^2^. These accessions had a median inflorescence density of 5 (out of 7). Images of the corresponding seed samples for each of these accessions are provided in **Figure S6 B, D–F**. Furthermore, plants presumed to be day-length sensitive did occasionally exhibit vegetative regrowth in the panicle, which Christiansen et al. (2010) also observed. The considerable variation in inflorescence traits, especially panicle size, is likely a result of the day-length sensitive accessions, which have values in contrast to those reported in the literature. For example, Bhargava et al. (2007b) report a much smaller range in inflorescence length (0.84 – 6.47 cm) and average inflorescence length (2.64 cm ± 0.24 standard errors of the mean). Manjarres-Hernández et al. (2021) reported a range in average inflorescence lengths from 39.0 – 72.4 cm. Given the conditions of this study, most plants produced a single inflorescence. This growth habit may differ from what would be observed if planted under field conditions, where multiple meristems could be produced. Despite the presence of accessions that exhibited sensitivity to day length, numerous accessions from the WCC could be valuable for breeding programs that wish make gains in reducing time to maturity under long-day conditions.

### 3.4 Yield and yield components

Yield and uniformity, as well time to maturity, are also important traits for long-day breeding programs (Zurita-Silva et al., 2014; Peterson et al., 2015). WSU VT entries had an average yield of 9.047 g/plant (sd = 1.825), compared to an average of 5.429 g/plant for WCC accessions (sd = 2.560). Average yield for WSU VT entries ranged from 7.105 g/plant (11WAQ-108.42) to 10.816 g/plant (11WAQ-102.8), while WCC accessions ranged from 0.989 g/plant (D-12021) to 10.645 g/plant (PI-634923). Certain studies report yield data on a per plant basis for field-grown germplasm that overlap to a certain extent with the accessions analyzed in this study. Bhargava et al. (2007b) report an average yield/plant of 16.27 g (± 2.06 standard errors of the mean) with a range from 1.29-39.39 g. Thiam et al. (2021) report yield values from 21.38 g/plant to 50.75 g/plant. Excluding an accession that did not produce any seed, average yield per plant values reported by Manjarres-Hernández et al. (2021) ranged from 12.28 g to 87.53 g.

Measured yield components included seed size (i.e. area), total seeds per plant, and TSW. WSU VT entries had an average seed area of 2.913 mm^2^ (sd = 0.276 mm^2^), an average of 2820 seeds per plant (sd = 657 seeds), and an average TSW of 3.269 g (sd = 0.506 g). Comparatively, WCC accessions had an average seed area of 2.507 mm^2^ (sd = 0.490 mm^2^), an average of 2118 seeds per plant (sd = 971 seeds), and an average TSW of 2.551 g (sd = 0.672 g) (data not shown). Overall, seed size, total seeds per plant, and TSW had a range of 2.765 mm^2^,6358 seeds, and 3.923 g, respectively (**Table 3**). The average TSW we report falls within the ranges reported for quinoa grown in Germany (1.2-3.3 g) (Präger et al., 2018), Italy (1.94-2.60 g) (De Santis et al., 2016), Pakistan (1.17 – 3.42) (Thiam et al., 2021), and India (0.78 – 4.09) (Bhargava et al., 2007). Präger et al. (2018) and Miranda et al. (2013) found that precipitation positively influenced TSW, which is an important consideration when interpreting the values reported in this study.

Certain accessions exhibited remarkable uniformity relative to others. For days to harvest and yield, CHEN-398 (sd=1 day) and D-12021 (0.403g/plant) had the minimum standard deviations, respectively. Alternatively, CHEN-430 (sd=38 days) and NSL-91567 (sd=3.083g/plant) had the maximum standard deviations for days to harvest and yield, respectively. Lack of uniformity could be related to high levels of heterozygosity within an accession. For example, Christensen et al. (2007) detected genetic heterogeneity in 32% of accessions at a given locus, suggesting that a considerable proportion of quinoa accessions present landraces or heterogenous seed lots. Uniformity, as well as plant height, can be especially important traits for mechanical harvesting, which the WSU quinoa breeding program primarily relies on. WSU VT entries had an average height at harvest of 113cm (sd = 15cm), compared to an average of 146cm (sd = 31) for the WCC accessions. In addition to days to harvest, average values for yield and height at harvest are indicators of targeted selection to overcome environmental and cropping system constraints within the WSU quinoa breeding program germplasm.

### 3.5 Protein quantity and quality

Perhaps just as important as yield, if not more important, is quinoa protein content and composition. Quinoa protein content can be highly variable and is often comparable to most cereals (Kozioł, 1992; Comai et al., 2007; Nowak et al., 2016). Overall, protein content had a mean of 13.99% (sd = 1.62%) and a range of 10.2% (8.46% - 18.69%), which is similar to the range (10.21% to 18.39%) reported by Rojas et al. (2015) for a Bolivian germplasm collection. In a review of quinoa nutritional composition, Nowak et al. (2016) found crude protein content to range from 9.1 – 15.1%. Increased nitrogen application can increase protein content in quinoa (Gomaa, 2013). We found that WSU VT entries had an average crude protein content of 12.20% (sd = 1.21%), compared to an average of 14.14% for the WCC accessions (sd = 1.56%) (data not shown). Various complex factors, such as site-specific environmental conditions and G×E interactions, can also influence protein content. Response to these factors may be accession dependent, as shown by Präger et al. (2018) and Miranda et al. (2013). However, Reguera et al. (2018) found that protein content significantly differed among, but not within, three different agroecological zones in a study using Salcedo-INIA, Titicaca, and Regalona. It is likely possible to develop broadly adapted germplasm with the capacity to maintain stable protein content in response to contrasting environmental conditions, as well as germplasm adapted to site-specific conditions such as low soil nitrate levels.

Protein quality can be defined based on protein digestibility values, which indicate the ease of absorption by the body, as well as amino acid content (Ruales and Nair, 1992; Nowak et al., 2016). Quinoa protein quality has garnered international attention due to the presence of all nine of the essential amino acids (EAA), leading to claims that quinoa is a complete protein. However, quinoa has been shown to have limiting amino acids, where content is insufficient to meet daily requirements (FAO/WHO/UNU, 2007). Daily requirements are established for several age groups, with infants having the highest requirements and adults having the lowest requirements. Therefore, if infant requirements are met, then requirements are met for all age groups. Conversely, if adult requirements are not met, then the requirements of any age group are not met. In our study, mean EAA values met adult requirements. However, mean values for leucine and lysine failed to meet infant requirements (**Table 6**). We found that approximately 40% (1028/2582) of samples failed to meet leucine, or leucine and valine requirements (0.46%) (12/2577), for any of the age groups (data not shown). Moreover, we found samples that failed to meet infant requirements for leucine, lysine, or valine requirements, or a combination of these amino acids and/or AAA, threonine, valine, and tryptophan requirements. We found two samples, representing replicates of the accessions Moroccan Yellow and Ames-13733, that met all EAA requirements for all age groups. Overall, approximately 48.6% (1273/2619) of the samples met adult requirements for all EAA. These results provide evidence that under certain conditions, quinoa samples (representing replicates of accessions), can have varying degrees of limiting EAA content.

**Table 6:** Mean and range, from minimum (min) to maximum (max), number of valid samples (*N*) for essential amino acids, and their respective adult and infant daily requirements.

Essential Amino Acids	*N*	Mean (Min - Max)	Adult Requirement^1^	Infant Requirement^1^
Histidine (mg g^-1^ protein)^ψ^	2617	27.28 (24.29-30.7)	15	20
Isoleucine (mg g^-1^ protein)^ψ^	2566	38.83 (33.62-43.43)	30	32
Leucine (mg g^-1^ protein)^ψ^	2634	59.36 (38.6-68.73)	59	66
Lysine (mg g^-1^ protein)^ψ^	2532	55.39 (46.59-65.06)	45	57
SAA (mg g^-1^ protein)^ψ^	2359	36.76 (28.55-41.88)	22	28
AAA (mg g^-1^ protein)^ψ^	2574	63.8 (47.43-70.68)	38	52
Threonine (mg g^-1^ protein)^ψ^	2619	31.38 (26.92-36.2)	23	31
Tryptophan (mg g^-1^ protein)^ψ^	2345	9.96 (7.87-12.22)	6	8.5
Valine (mg g^-1^ protein)^ψ^	2577	44.39 (37.52-51.28)	39	43

ψ = fixed at 14% moisture content; SAA, sulfur amino acids; AAA, aromatic amino acids.

^1^adapted from WHO/FAO/UNU (2007) suggested indispensable amino acid requirements.

BLUEs provided insight into which accessions may have the potential to satisfy daily requirements, and which accessions may fail to meet daily requirements (**Supplementary Table S5**). The BLUEs indicate that 154 accessions fail to meet leucine requirements for any of the age groups, including 5 VT entries and 149 WCC accessions. This is the only limiting amino acid when comparing to adult requirements. When considering infant requirements, all accessions had insufficient leucine content. Moreover, certain accessions failed to meet various combinations of leucine, lysine, threonine, valine and tryptophan infant requirements. We found 53 accessions (all WCC accessions) that had limiting amino acid content for four amino acids (leucine, lysine, threonine, and valine or tryptophan). Of the 76 accessions limited solely by leucine content, 24 were VT entries and 52 were WCC. These accessions may be especially useful to producers interested in quinoa with the potential to produce exceptional protein quality. Breeding programs may also be interested in further examining these accessions, especially if they aim to increase leucine content and endeavor to meet all EAA requirements for all age groups.

Präger et al. (2018) reported amino acid values for four accessions, two of which were included in this study (Titicaca and Puno), grown over two years in southwestern Germany at one location with comparable day-length conditions to those applied in this study. In the first year of their study, mean values for Puno met isoleucine, sulfur amino acids (methionine and cysteine; SAA), aromatic amino acids (phenylalanine and tyrosine; AAA), threonine and tryptophan requirements for all age groups, while mean values for Titicaca met tryptophan and valine requirements for all age groups. In the second year, mean values for Puno met SAA, AAA, threonine, tryptophan and histidine requirements for all age groups, while mean values for Titicaca met SAA, tryptophan, valine and histidine requirements for all age groups. Across both years, values reported by Präger et al. (2018) for each of the varieties studies failed to leucine requirements for any age groups. In our study, we found that mean values for Titicaca (59.67 mg/g protein) (both VT and WCC source) and Puno (60.01 mg/g protein) failed to meet only the leucine requirements for all age groups. The differences observed between these studies could be due to numerous factors, but is most likely a result of differing climatic and soil conditions and complex accession-by-environment interactions impacting amino acid content (Thanapornpoonpong et al., 2008; Varisi et al., 2008; Gonzalez et al., 2012; Geren, 2015; Bascuñán-Godoy et al., 2016; Reguera et al., 2018). However, these mechanisms are still not well understood in quinoa.

In a study of diverse quinoa germplasm, Craine & Murphy (2020) report samples that failed to meet leucine, lysine and tryptophan requirements for all age groups. Miranda et al. (2012) reported values for six accessions that failed to meet the lysine requirements for all age groups, and one accession that failed to meet the leucine requirements for all age groups. The mean values reported by Nowak et al. (2016) failed to meet valine requirements for all age groups, in addition to not meeting the isoleucine, lysine and leucine requirements for infants. In their study, methionine (88 mg/g protein), the aromatic amino acids (AAA) (76 mg/g protein), and leucine (71 mg/g protein) had the largest range in values. We found leucine to have the largest range (30.1 mg/g protein), followed by the AAA (23.3 mg/g protein) and lysine (18.5 mg/g protein). Given these large ranges, there is considerable variation among quinoa germplasm for essential amino acid content, which can result in insufficient content to meet daily requirements. Regarding leucine and lysine content, larger maximum values reported by Nowak et al. (2016) for leucine (94 mg/g protein) and lysine (78 mg/g protein), compared to what we found for leucine (68.7 mg/g protein) and lysine (65.0 mg/g protein), indicates that sources of variation exist that could contribute to higher content for these limiting amino acids. Granado-Rodríguez et al. (2021a) found the crop year to be a determining factor for content of all amino acids, while genotype only impacted certain amino acids, and their interaction only impacts aspartic acid, cysteine, and arginine. Furthermore, they found that all samples analyzed met lysine and leucine daily requirements for all age groups, while only certain samples met tryptophan and sulfur amino acid requirements. Their results provide additional evidence indicating that quinoa can have limiting amino acid content. Through proper management and breeding for improved lysine and sulfur amino acid content, it may be possible to realize the potential of quinoa to consistently meet daily essential amino acid requirements for all age groups.

### 3.6 Strategies for germplasm improvement

**Figure 4:**
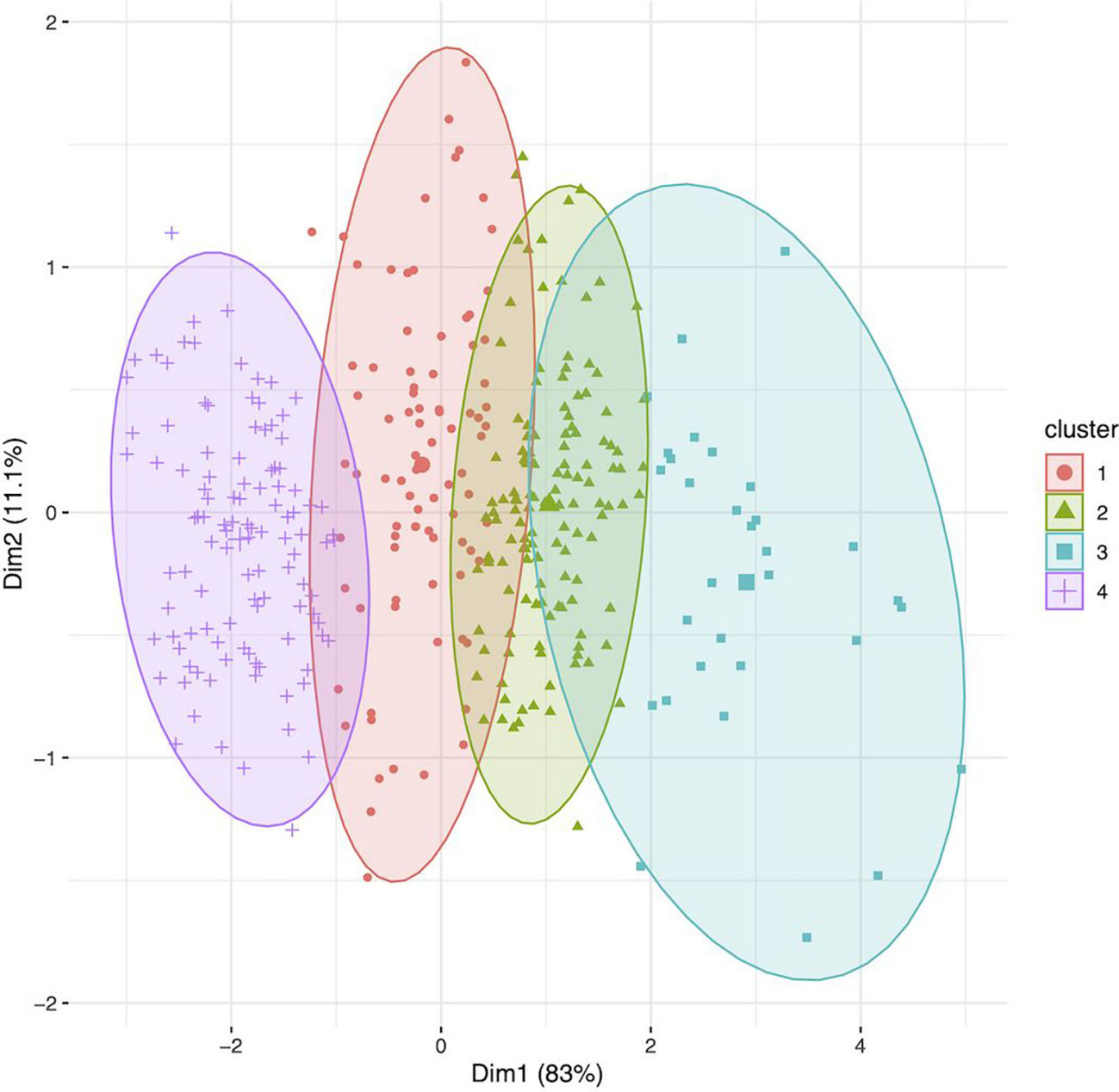
Clustering (K-means; groups = 4) of the genotypes (n = 360) using data for days to harvest, height at harvest, and yield/plant. Cluster identity for each accession is provided in **Supplementary Table S4** under column 3TC (i.e. three trait cluster), and passport data for each accession is provided in **Supplementary Table S1**. Cluster 4 includes all the variety trial (VT) entries (*N = 26*) from the Washington State University (WSU) quinoa breeding program, along with 86 accessions from the world core collection (WCC). Clusters 1, 2, and 3 were comprised of accessions from the WCC. When compared to the mean value for VT entries, 14 accessions in cluster 4 had shorter mean days to harvest, 35 accessions from cluster 4 and 3 accessions from cluster 3 had shorter average height at harvest; and 16 accessions from cluster 4 had greater average yield per plant. Seven WCC accessions satisfied each of these conditions. These accessions include PI-634923, PI-614889 (a parent of most of the WSU experimental lines), D-11889, Titicaca (WCC seed source), Vikinga, BO-3 and Moroccan Yellow.

The World Core Collection has the potential to provide a valuable influx of germplasm into long-day breeding programs. In the context of environmental conditions, such as heat stress, fewer days to harvest may allow quinoa to escape negative impacts on growth and reproduction. For example, Matías et al. (2021) demonstrate that in a two-year study, with elevated temperatures and lower relative humidity in one year, that varieties with a shorter cycle (i.e. fewer days to harvest) had greater harvest index (i.e. higher yield relative to vegetative biomass). Clustering of the WSU VT entries and WCC accessions using days to harvest, plant height at harvest, and yield revealed congruencies between the two collections. With K-means clustering fixed at five groups, all of the VT entries clustered independently from the WCC accessions. After adjusting the number of groups to four, 86 accessions from the WCC clustered with the VT entries in cluster 4 (**Figure 4**). The identity of each accession in each cluster is provided in **Supplementary Table S4**. Of the WCC accessions included in cluster 4, 1 originated from Switzerland, 1 originated from Bolivia, 2 originated from the UK, 2 originated from Denmark, 3 originated from Argentina, 3 originated from the US, 16 originated from seeds donated to the USDA by Emigdio Ballon in New Mexico, US, although they are not native to New Mexico, US, 30 originated from Chile, and 26 are of unknown origin. Sorting the WCC accessions for each trait revealed which accessions had an average trait value greater than the mean value for the VT entries. For example, 14 accessions in cluster 4 had mean days to harvest shorter than the VT mean, 35 accessions from cluster 4 and 3 accessions from cluster 3 had an average height at harvest shorter than the VT mean, and 16 accessions from cluster 4 had an average yield greater than the VT mean. Seven WCC accessions satisfied each of these conditions. These accessions include PI-634923, PI-614889 (a parent of most of the WSU experimental lines), D-11889, Titicaca (WCC seed source), Vikinga, BO-3 and Moroccan Yellow. While these accessions may represent the most promising germplasm for long-day breeding programs to utilize, in addition to the VT experimental lines that have been selected under such conditions, they represent a fraction of the many possibly accessions to consider. Accessions from the WCC potentially harbor other useful traits besides those selected for the cluster analysis.

**Figure 5:**
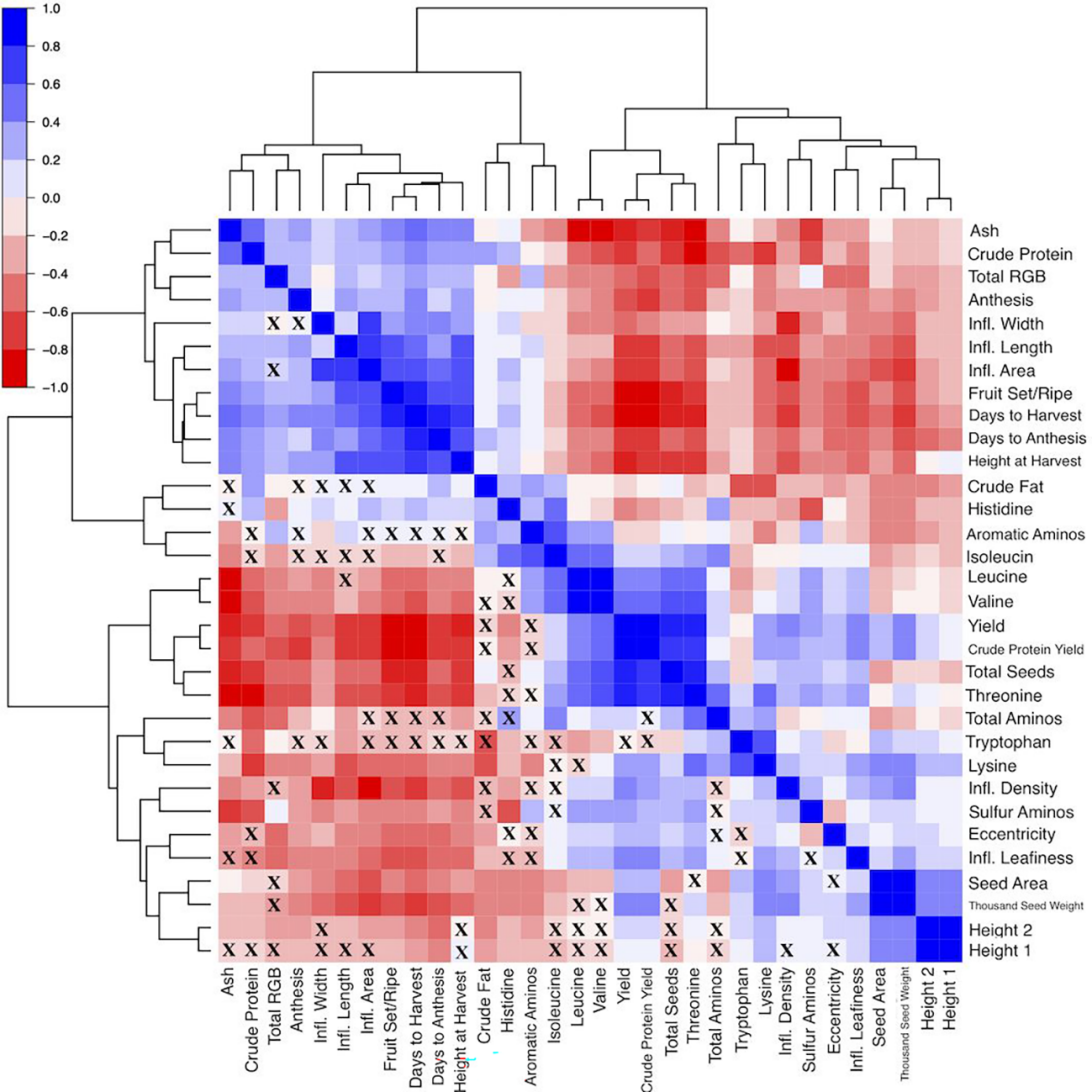
Heat map visualization of the correlation matrix, where Spearman correlation coefficients range from -1 (solid red fill) to 1 (solid blue fill). The dendrogram is constructed using hierarchical clustering of the variables included on the edges. Correlations that are not significant (p > 0.05) are denoted by an “X” in the corresponding cell. Note: DTA, Days to Anthesis; Anth, Anthesis (days); FSR, FruitSet/Ripening (days); DTH, Days to Harvest; Ht_1, Plant Height-5wk After Sowing (cm); Ht_2, Plant Height-6wk After Sowing (cm); Ht_Harv, Plant Height-Harvest (cm); Infl_L, Inflorescence Length (cm); Infl_W, Inflorescence Width (cm); Infl_Area, Inflorescence Area (cm); Infl_Density, Inflorscence Density; Infl_Leafiness, Inflorescence Leafiness; Ash, Ash Content;CF, Crude Fat Content;CP, Crude Protein Content;TAA, Total Amino Acid Content;His, Histidine Content; Ile, Isoleucine Content; Leu, Leucine Content; Lys, Lysine Content; SAA, SAA Content; AAA, AAA Content; Thr, Threonine Content; Trp, Tryptophan Content; Val, Valine Content; CP Yield, Crude Protein Yield (g plant-1); Yield, Yield (g plant-1); TSW, Thousand Seed Weight (g); Seeds, Total Seeds per plant; Area, Seed Area (mm2); Eccen, Seed Eccentricity; sumRGB, Total Red, Green, Blue.

Measuring a large number of yield-related traits including seed morphology enabled an analysis of correlations that could aid simultaneous trait improvement. In general, the phenotypic traits formed two groups (**Figure 5**). The first group consisted of thousand seed weight, seed area, height at 5 weeks and 6 weeks after sowing, seed shape (i.e. seed eccentricity), yield and protein yield, total amino acids, the sulfur amino acids, lysine, tryptophan, threonine, leucine, and valine. This group of traits had both positive and negative correlations existing both among the traits in the groups, and with the traits in the second. The second group consisted of the phenological growth stages (anthesis, days to anthesis, fruit set/ripening, days to harvest), seed composition traits (crude protein, ash, and crude fat), seed color (i.e. sum of red, green, blue values), inflorescence size (length, width and area), and height at harvest. The second group of traits generally had positives relationships with one another. These relationships may assist breeders in prioritizing phenotyping efforts, by indicating which traits may be the most useful to focus their efforts on.

In the context of long-day breeding programs, reducing days to harvest may impact several other traits. We found that days to harvest had strong positive correlations with days to anthesis, fruit set/ripening, and plant height (**Figure 5**; **Supplementary Table S3**). Breeders may still decide to record plant height at harvest and phenological growth stages besides days to harvest, especially if stressors like heat are expected to be present during critical reproductive stages (e.g. flowering and milk grain stage) (Geerts et al., 2008; Hinojosa et al., 2019; Alvar-Beltrán et al., 2020; Tovar et al., 2020). High-throughput methods may increase the efficiency of phenotyping these traits (Madec et al., 2017; Zhang et al., 2020), and methods are being developed specifically for quinoa (Stanschewski et al., 2021). Regarding seed composition traits, days to harvest had moderate positive correlations with crude protein content, ash, and a weak positive correlation with crude fat. Storage proteins increase significantly in quinoa during maturation, in addition to oil accumulation, which provides evidence of a possible tradeoff between maturation and seed composition (Shen et al., 2022). For instance, Grimberg et al. (2022) found Pasankalla matured 13 days after Titicaca and Regalona under controlled conditions (12-hour days) and had significantly higher protein and oil content.

Days to harvest also had moderate positive correlations with inflorescence length, width, and area. In general, later maturing plants had larger panicles. Moreover, inflorescence density and leafiness had moderate negative correlations with days to harvest (**Figure 5**; **Supplementary Table 3**). This relationship seems to provide additional evidence for a distinctive day-length sensitive phenotype. However, this phenotype may be an artifact of ecotype origin (i.e. altiplano types leafy panicles independent of photoperiod sensitivity). Under certain field conditions, a lax, lower density panicle may facilitate maturation by increasing airflow and reducing water-holding capacity, which could also reduce the risk of preharvest sprouting (PHS) and yield loss. Examples of WSU VT entries, selected for PHS resistance, that exhibit this phenotype are shown in **Figure S7**. Analysis of quinoa inflorescence images, which is currently under development, may provide a higher-throughput method to quantify inflorescence size as well as inflorescence characteristics such as color, shape, density and leafiness, which could be deployed in various settings. Breeders may benefit from understanding these phenotypes, as they can be useful for characterizing germplasm and may contribute a better understanding of how inflorescence traits may influence yield.

Yield is a relatively simple trait to measure and is often considered to be of the paramount importance. We found a moderate negative correlation between panicle length and yield (*r* = -0.57) (**Supplementary Table S3**). Manjarres-Hernández et al. (2021) found a similar relationship (*r* = -0.51), and the result of shorter panicles producing more seeds is contrary to results of De Santis et al. (2018) and Maliro et al. (2017). Yield, TSW, total seeds per plant, and seed area had weak positive correlations with inflorescence density and leafiness, which provides evidence of potential minor contributions from inflorescence traits to yield and yield components (**Figure 5**; **Supplementary Table S3**). Overall, late maturing plants generally had lower yield, as evidenced by a moderate negative correlation. Furthermore, late maturing plants generally had smaller seed size and lower TSW. While these seed traits require more complex and time-consuming methods to properly measure, they are important for understanding the relative contributions of yield components. Yield had a weak negative correlation with seed color. Seed color is defined in this context using the RGB color model, which is an additive color model. For example, the color white has RGB values of (255, 255, 255). Plants that had seed color values closer to white generally had lower yield. Total seeds per plant had a strong positive correlation with yield, compared to moderate positive correlations between both seed size and TSW with yield. Thiam et al. (2021) found yield harvest index, and thousand kernel weight to be the main variables that are positively correlated. Larger seeds generally had higher TSW and yield, a result also found by Präger et al. (2018) and Manjarres-Hernández et al. (2021). Therefore, it may be possible to rely on seed size measurements or another method, such as test weight, if resources are limited and quantifying TSW is impractical. However, a relatively stronger relationship between total seeds per plant and yield, than between seed area and TSW with yield, warrants further research to examine the potential of per plant seed production to improve yield over increasing seed size or weight.

**Figure 6:**
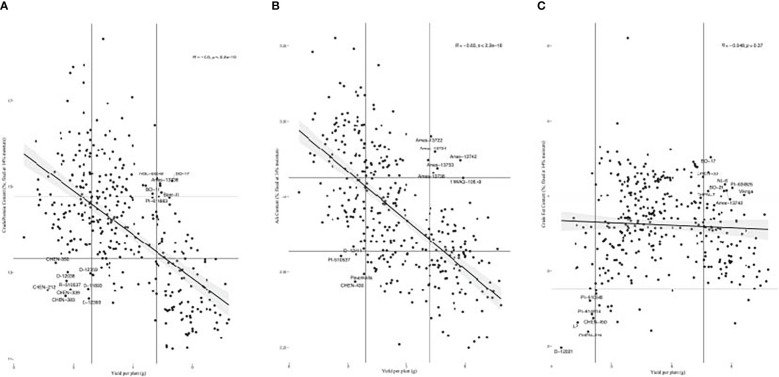
**(A)** Scatter plot of accession best linear unbiased estimate values for crude protein content by yield per plant. Accessions in the top 30% or bottom 30% of values for both traits are labeled, as shown within the vertical and horizontal lines at the corresponding quantiles. **(B)** Scatter plot of accession best linear unbiased estimate values for ash content by yield per plant. Accessions in the top 30% or bottom 30% of values for both traits are labeled, as shown within the vertical and horizontal lines at the corresponding quantiles. **(C)** Scatter plots of accession best linear unbiased estimate values for crude fat content by yield per plant. Accessions in the top 25% or bottom 5% of values for both traits are labeled, as shown within the vertical and horizontal lines at the corresponding quantiles.

We found varying relationships between yield and the seed composition traits. These traits included crude protein, crude fat, ash and total amino acid content (**Figure 5**; **Supplementary Table S3**). Among these traits, crude protein, total amino acid content, and ash content had strong positive correlations with one another, while crude protein had a moderate positive correlation with crude fat content. In a study of Pasankalla, Titicaca, and Regalona, Grimberg et al. (2022) found Pasankalla to have the highest protein and oil content. These results suggest that protein and oil content could be enhanced simultaneously and could be aided by increasing the embryo portion of seeds where these components are concentrated (Burrieza et al., 2014; Gargiulo et al., 2019). Conversely, yield had a strong negative correlation with amino acid and ash content and had a moderate negative correlation with crude protein content. However, there are examples of accessions that had relatively high yield and relatively high content of the seed components. For instance, BO-17, Baer-II, Ames-13738, BO-11, and NSL-NSL-86649 had BLUEs for crude protein content and yield in the top 30% of all accessions (**Figure 6A**). Moreover, Ames-13722, Ames-13734, Ames-13742, Ames-13753, Ames-13738 had BLUEs for ash content and yield in the top 30% of all accessions (**Figure 6B**), while BO-17, CHEN-33, BL-6, PI-634925, BO-29, CHEN-7, Ames-13742, and Vikinga had yield and crude fat values in the top 25% of all accessions (**Figure 6C**). The combination of crude protein and yield is captured by the crude protein yield trait. Crude protein yield has an extremely strong positive relationship with seed yield, and a moderate negative relationship with crude protein content (**Figure 5**; **Supplementary Table S3**). Therefore, crude protein yield is positively correlated with many of the traits that yield has positive relationships with, such as height at 5 and 6 weeks after sowing, inflorescence density and leafiness, certain essential amino acids (e.g. leucine, lysine, sulfur amino acids), and total seeds per plant. We found weak correlations between the seed morphology and composition traits, indicating that measurement of the former may not be a reliable indicator of the latter. Average area had a weak negative correlation with crude fat content and TSW had weak negative correlations with each of the seed composition traits (**Figure 5**; **Supplementary Table S3**).

These results indicate that solely focusing on increasing seed yield may present the risk of inadvertently diminishing quinoa nutritional quality. To reduce this risk, specific attention should be given to phenotyping and monitoring the content of important seed components. The WSU quinoa breeding program has developed a high-throughput system for phenotyping seed components and morphology to accomplish this goal.

Standard heritability values ranged from 0.68 (anthesis) to 0.99 (total red, green and blue) (**Table 7**). Considering heritability calculated on a genotype-difference basis (i.e. ${\text{H}}_{\mathrm{Cullis}}^{2}$ ), all traits had values greater than or equal to 0.90, except for anthesis, inflorescence leafiness, leucine continent, aromatic amino acid content, plant height 5 weeks after sowing, valine content, and total amino acid content. Hafeez et al. (2022) report comparable broad-sense heritability values for yield, plant height, inflorescence length, seed number, and the phenological growth stages, and a lower value for TSW. With data from the F3 of a biparental population from PI-614889 (female parent) and CHEN-109 (male parent), grown under long-day field conditions, Maldonado-Taipe et al. (2022) report lower broad-sense heritability values for plant height and panicle length, possible from a wide range and large variability in the male parent, and comparable values for days to anthesis, days to flowering, panicle density, and thousand seed weight. For traits with high heritability values (i.e. greater than 0.90), selection within the world core collection would likely result in a rapid advance in trait values relative to those observed for the collection. However, gains would likely decrease rapidly following initial rounds of selection. We found that total red, green and blue values had the largest ratio between the genotypic and the residual variance components, followed by days to harvest, seed area, days to anthesis, threonine content, and thousand seed weight (**Table 7**).

**Table 7:** Variance components for each trait, including accession by greenhouse interaction ( $\sigma_{\text{g}}^{2}$ x gh), accession ( $\sigma_{\text{g}}^{2}$ ), replicate ( $\sigma_{\text{rep}}^{2}$ ), greenhouse ( $\sigma_{\text{gh}}^{2}$ ), residual ( $\sigma_{\text{err}}^{2}$ ), and the ratio of accession variance to residual variance ( $\sigma_{\text{gh}}^{2}$ : $\sigma_{\text{err}}^{2}$ ), in addition to Cullis heritability ( ${\text{H}}_{\mathrm{Cullis}}^{2}$ ) and standard heritability ( ${\text{H}}_{\mathrm{Standard}}^{2}$ ).

Traits	$\sigma_{\text{gh}}^{2}$	$\sigma_{\text{g}}^{2}$	$\sigma_{\text{g x gh}}^{2}$	$\sigma_{\text{rep}}^{2}$	$\sigma_{\text{err}}^{2}$	$\sigma_{\text{g}}^{2}$ : $\sigma_{\text{err}}^{2}$	${\text{H}}_{\mathrm{Cullis}}^{2}$	${\text{H}}_{\mathrm{Standard}}^{2}$
Days to Anthesis	8.4	46.3	1.6	5.9	15.6	2.9	1.0	0.9
Anthesis (days)	0.7	3.9	6.4	0.3	26.2	0.2	0.7	0.5
FruitSet/Ripening (days)	0.0	93.0	27.5	0.6	75.0	1.2	0.9	0.9
Days to Harvest	9.1	354.6	32.4	7.9	109.1	3.3	1.0	0.9
Plant Height-5wk After Sowing (cm)	0.0	13.4	1.4	15.5	23.2	0.6	0.9	0.8
Plant Height-6wk After Sowing (cm)	0.0	33.3	2.4	32.0	35.2	0.9	0.9	0.9
Plant Height-Harvest (cm)	0.0	666.1	42.7	12.5	269.1	2.5	1.0	1.0
Inflorescence Length (cm)	1.6	77.2	15.1	0.8	45.5	1.7	0.9	0.9
Inflorescence Width (cm)	0.0	10.0	0.0	0.2	11.7	0.9	0.9	1.0
Inflorescence Area (cm)	135.1	25283.4	3294.2	504.1	18552.3	1.4	0.9	0.9
Inflorescence Color	0.1	6.4	1.1	0.0	7.0	0.9	0.9	0.9
Inflorescence Shape	0.0	0.4	0.1	0.0	0.4	0.9	0.9	0.9
Inflorescence Density	0.1	1.3	0.2	0.0	1.3	1.0	0.9	0.9
Inflorescence Leafiness	0.0	0.9	0.3	0.0	1.6	0.5	0.9	0.8
Ash Content (%)	0.0	1.6	0.2	0.1	1.0	1.5	0.9	0.9
Crude Fat Content (%)	0.0	0.0	0.0	0.0	0.0	1.1	0.9	0.9
Crude Protein Content (%)	0.0	0.2	0.0	0.0	0.2	1.0	0.9	0.9
Total Amino Acid Content (mg g^-1^ protein)^ψ^	0.0	178.5	33.5	5.9	215.8	0.8	0.9	0.9
Histidine Content (mg g^-1^ protein)^ψ^	0.0	0.3	0.0	0.0	0.2	1.6	0.9	1.0
Isoleucine Content (mg g^-1^ protein)^ψ^	0.0	0.6	0.1	0.1	0.7	0.8	0.9	0.9
Leucine Content (mg g^-1^ protein)^ψ^	0.0	2.0	0.4	0.4	3.2	0.6	0.9	0.9
Lysine Content (mg g^-1^ protein)^ψ^	0.0	5.4	0.2	0.1	2.4	2.3	1.0	1.0
SAA Content (mg g^-1^ protein)^ψ^	0.0	1.2	0.1	0.1	0.9	1.3	0.9	1.0
AAA Content (mg g^-1^ protein)^ψ^	0.0	1.5	0.2	0.1	2.2	0.7	0.9	0.9
Threonine Content (mg g^-1^ protein)^ψ^	0.0	1.5	0.1	0.0	0.6	2.6	1.0	1.0
Tryptophan Content (mg g^-1^ protein)^ψ^	0.0	0.2	0.0	0.0	0.2	1.1	0.9	0.9
Valine Content (mg g^-1^ protein)^ψ^	0.0	1.4	0.3	0.2	1.5	0.9	0.9	0.9
Protein Yield (g plant^-1^)	0.0	0.1	0.0	0.0	0.0	1.4	0.9	0.9
Yield (g plant^-1^)	0.1	5.0	0.6	0.0	2.3	2.2	1.0	0.9
Thousand Seed Weight (g)	0.0	0.3	0.0	0.0	0.1	2.5	1.0	1.0
Total Seeds (seeds plant^-1^)	6958.1	534459.3	62183.4	2431.3	326215.7	1.6	0.9	0.9
Seed Area (mm^2^)	0.0	0.2	0.0	0.0	0.1	3.0	1.0	1.0
Seed Eccentricity	0.0	0.0	0.0	0.0	0.0	1.8	1.0	0.9
Total Red, Green, Blue	0.0	2469.3	26.7	10.3	421.2	5.9	1.0	1.0

### 3.7 Caveats, limitations, and future directions

Given the long-day photoperiod used in this study, results and discussion focuses on potential implications for long-day quinoa breeding programs. Attention is given to comparisons between the World Core Collection and the WSU Quinoa Breeding Program variety trial (VT) entries. The VT entries represent varieties and breeding lines adapted to the long-day conditions and agroecosystems of the Pacific Northwest region of the United States. Given this specific context, it would be especially useful for a similar study to be replicated under short-day conditions to compliment this study. Together, these studies would provide a more complete evaluation of the world core collection. Moreover, this would provide a better indication of the potential of the world core collection to benefit producers and breeders across varying environments, depending on day-length conditions. The delay in sowing dates for the two greenhouses is important to note as a potential source of variation, which could potentially impact the trial results and robustness of comparing accessions between the two greenhouses. Perhaps most importantly, the controlled, greenhouse conditions of this study cannot be assumed to be reproducible under field conditions. Myriad factors can influence plant growth and reproduction, such as photosynthetically active radiation, temperature, soil moisture, and humidity, and are likely to be in greater flux outside of the controlled environment employed in this study. The world core collection must also be screened under field conditions and, which will provide a more realistic indication of its potential utilization. Such a study has been completed by Patiranage et al. (2020) for the WCC accessions also evaluated in this study. A study of quinoa nutritional quality by Granado-Rodríguez et al. (2021b) indicates how genotypes respond to environmental factors and their influence on seed nutritional quality and provides evidence for significant effect of cropping year. Therefore, evaluation across multiple years is necessary.

Most, but not all of the WCC and VT accessions used in this study are publicly available for research. The named varieties Vikinga, Puno, and Titicaca were developed by Quinoa Quality, a private seed company, and have restricted availability (**Supplementary Table S1**). Access to these materials for research or commercial applications requires direct inquiry to Quinoa Quality (https://www.quinoaquality.com). Several of the other named varieties used, such as Redhead and Cherry Vanilla, were developed by Frank Morton at Wild Garden Seed in Philomath, Oregon, USA, and were released with an Open Source Seed Pledge to maintain them perpetually free of any intellectual property restrictions and publicly available (Kloppenburg, 2014; OSSI, 2014; Luby et al., 2015; Luby and Goldman, 2016). Seed of these varieties are available to purchase from Wild Garden Seed (https://www.wildgardenseed.com). WSU accessions are available for research in small quantities depending on seed availability and the completion of a material transfer agreement (MTA). WSU intends to publicly release one or several of these experimental lines within the next few years, which should provide increased supply and distribution of the lines released. Publicly available WCC accessions are likewise available depending on seed availability by contacting researchers at KAUST, namely Dr. Mark Tester, and the completion of an MTA. WCC accessions with a PI- or an Ames- prefix are also available from the USDA NPGS in small quantities (200 seeds) to researchers across the globe and can be searched at: https://npgsweb.ars-grin.gov/gringlobal/search.

With further study of the germplasm evaluated in this study, under different agroecological conditions and across multiple environments and crop years, trait variability and relationships will be better understood. Ultimately, expanding the understanding of quinoa genetic diversity with respect to important phenotypes as demonstrated in this study will be provide a solid foundation for breeding and development of improved germplasm.

## 4 Conclusion

We observed considerable variation existed among the quinoa accessions in the world core collection. Days to harvest, yield/plant, fruit set/ripening, days to anthesis, height at harvest, inflorescence area, TSW, inflorescence length, and total amino acid content contributed the most to the first principal component, which explained 39.8% of the total variation. The presence of day-length sensitivity accessions likely contributed to the extreme variation observed for days to harvest. These plants tended to have a phenotype characterized by greater height at harvest, larger panicle length, width and area, lower seed yield, and higher seed composition content. Principal component and cluster analysis illustrated how the germplasm could be separated according to the analyzed traits into four groups with unique variability. Group 1 represented accessions that may be the most relevant to long-day breeding programs, with accessions likely representing germplasm originating from higher latitudes in central and southern Chile (approximately 34-40°S). Group 1 was characterized by fewer days to harvest, lower height at harvest, and higher yield. However, this group did have lower protein and ash content than group 4, which likely represented accessions from the Highland germplasm pool originating from lower latitudes in Peru and Bolivia. We found that days to harvest had moderate negative correlations with yield, TSW and seed area, and moderate positive correlations with height at harvest, inflorescence area, crude protein and ash content. Yield had moderate positive correlations with seed size and TSW, and negative correlations with total amino acid, crude protein, and ash content. These results indicate that improvements to yield in quinoa must be made while simultaneously monitoring seed composition, to avoid selecting against nutritional quality in pursuit of higher yields. Using BLUEs, we provide insight into which accessions may have the most promising assembly of trait values. Overall, this study provides a much-needed phenotypic characterization of a diverse collection of accessible quinoa accessions, and provides insights into phenotypic relationships, which together will assist breeders in developing germplasm for novel production regions.

## Data availability statement

The original contributions presented in the study are included in the article/**Supplementary Material**. Further inquiries can be directed to the corresponding author.

## Author contributions

EC and KM designed the study with MT. MT and SS provided the germplasm. EC and AD carried out the study and phenotyping, with assistance from DP and supervision from KM. NM and ES designed the All Grains tool for seed digital image analysis. EC performed the statistical analyses and wrote a draft of the manuscript. EC revised the manuscript with feedback from AD, DP, NM, SS, ES, MT and KM. KM and MT secured funding. All authors contributed to the article and approved the submitted version.
