# Supplementary material for: A comprehensive characterization of agronomic and end-use quality phenotypes across a quinoa world core collection
Source: Front Plant Sci. 2023 Feb 16;14:1101547. doi: 10.3389/fpls.2023.1101547 (PMC9978749; doi:10.3389/fpls.2023.1101547)
Supplement: Supplementary file 2 [file Presentation_2.zip › Presentation 2 updated/SM Figure S2.PDF]

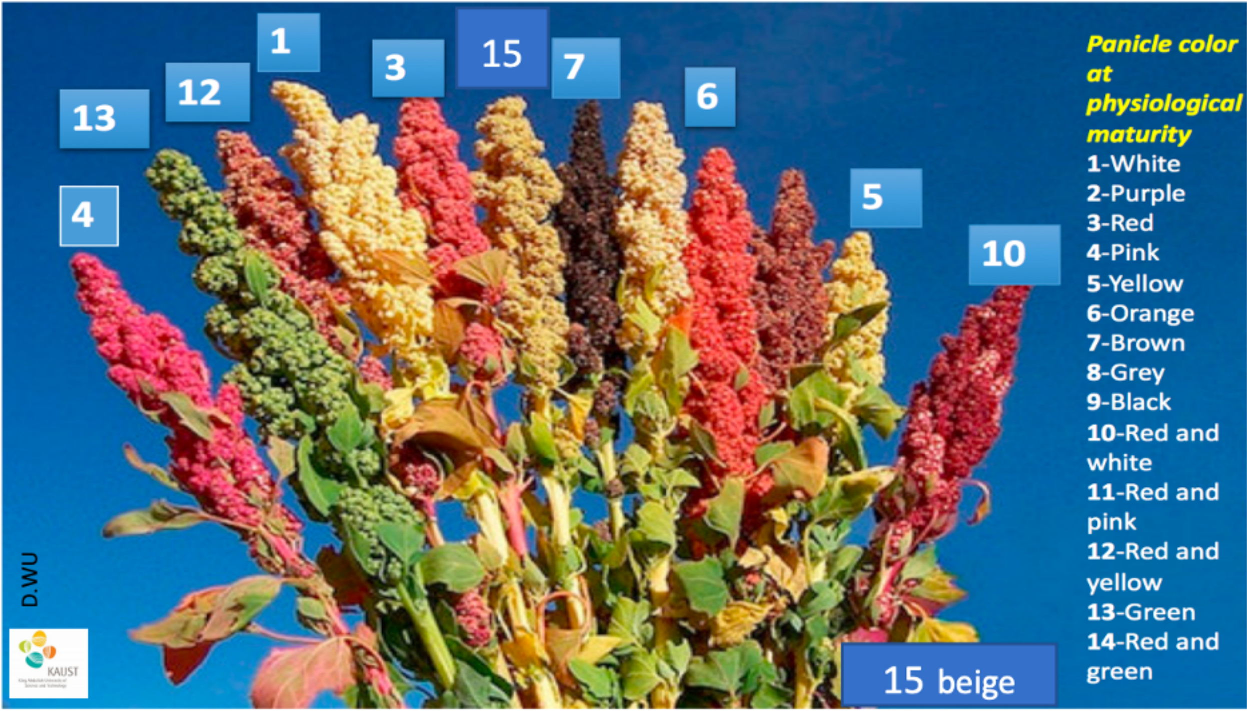

1
15
3
7
12
13
6
4
5
10
15 beige
Panicle color at physiological maturity
1-White
2-Purple
3-Red
4-Pink
5-Yellow
6-Orange
7-Brown
8-Grey
9-Black
10-Red and white
11-Red and pink
12-Red and yellow
13-Green
14-Red and green
D.WU
KAUST
