# Supplementary figures and images for: A comprehensive characterization of agronomic and end-use quality phenotypes across a quinoa world core collection
Source: Front Plant Sci. 2023 Feb 16;14:1101547. doi: 10.3389/fpls.2023.1101547 (PMC9978749; doi:10.3389/fpls.2023.1101547)

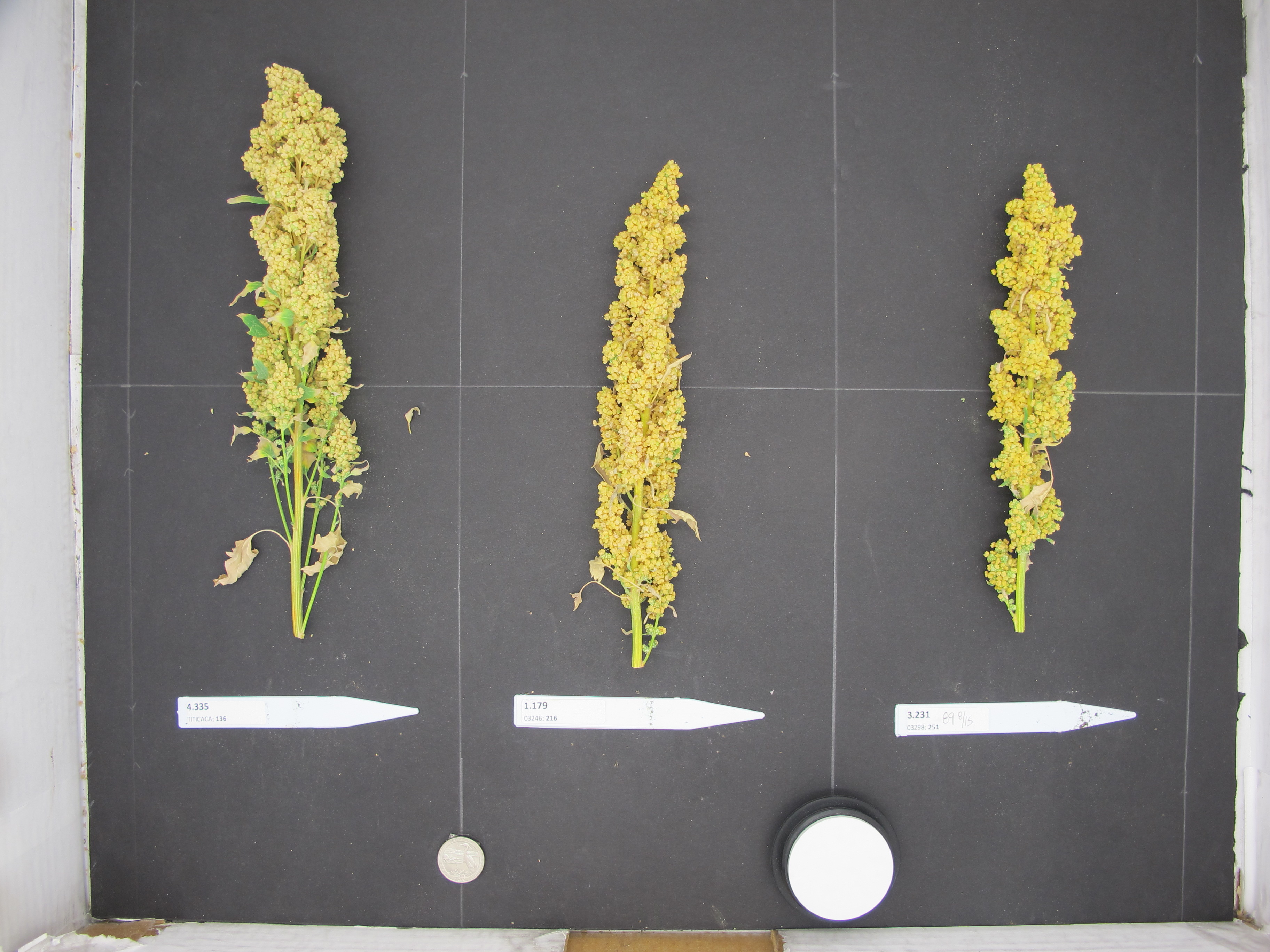

Supplement: Supplementary file 2 [file Presentation_2.zip › Presentation 2 updated/SM Figure S6C.JPEG]

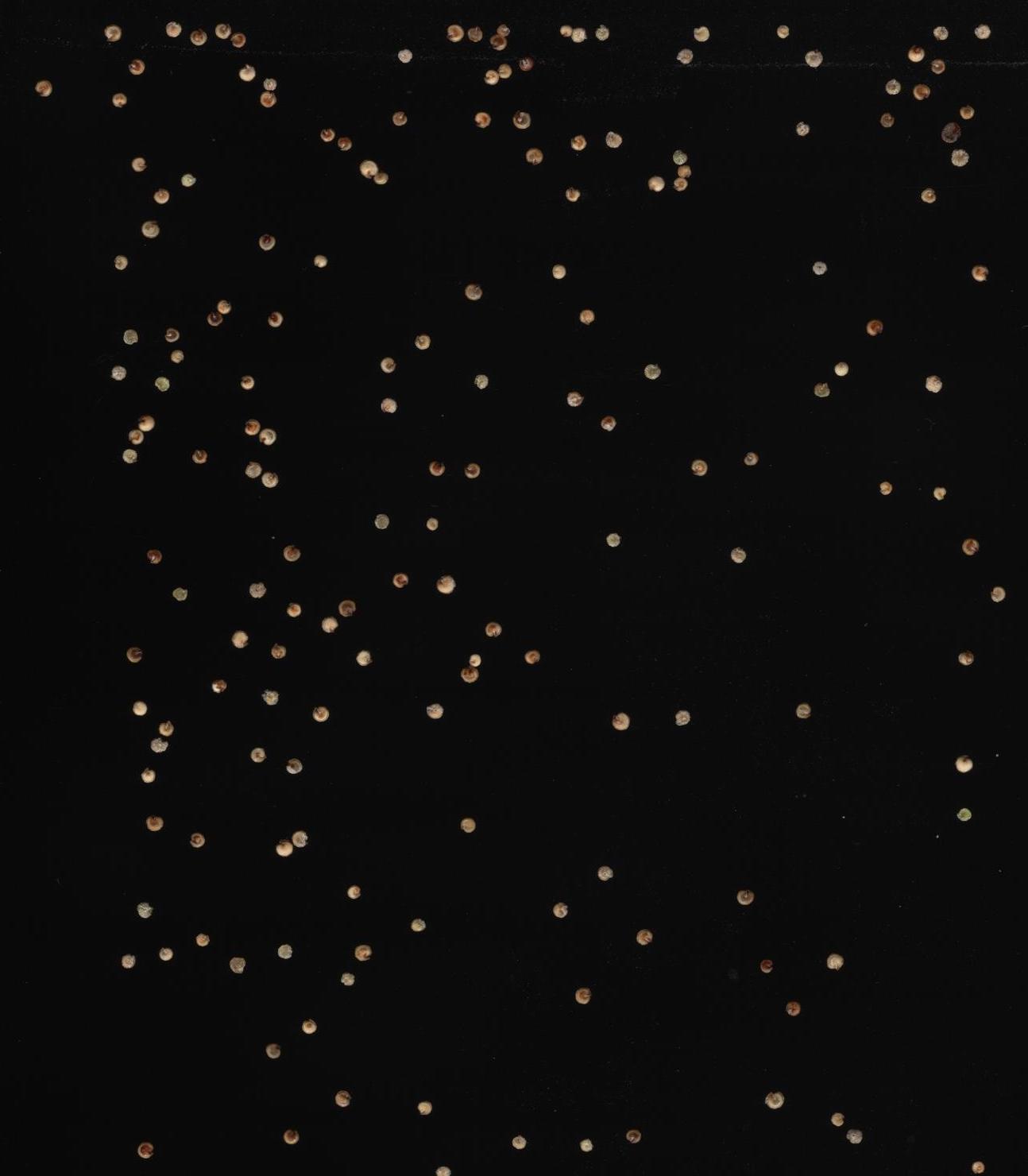

Supplement: Supplementary file 2 [file Presentation_2.zip › Presentation 2 updated/SM Figure S6B.JPEG]

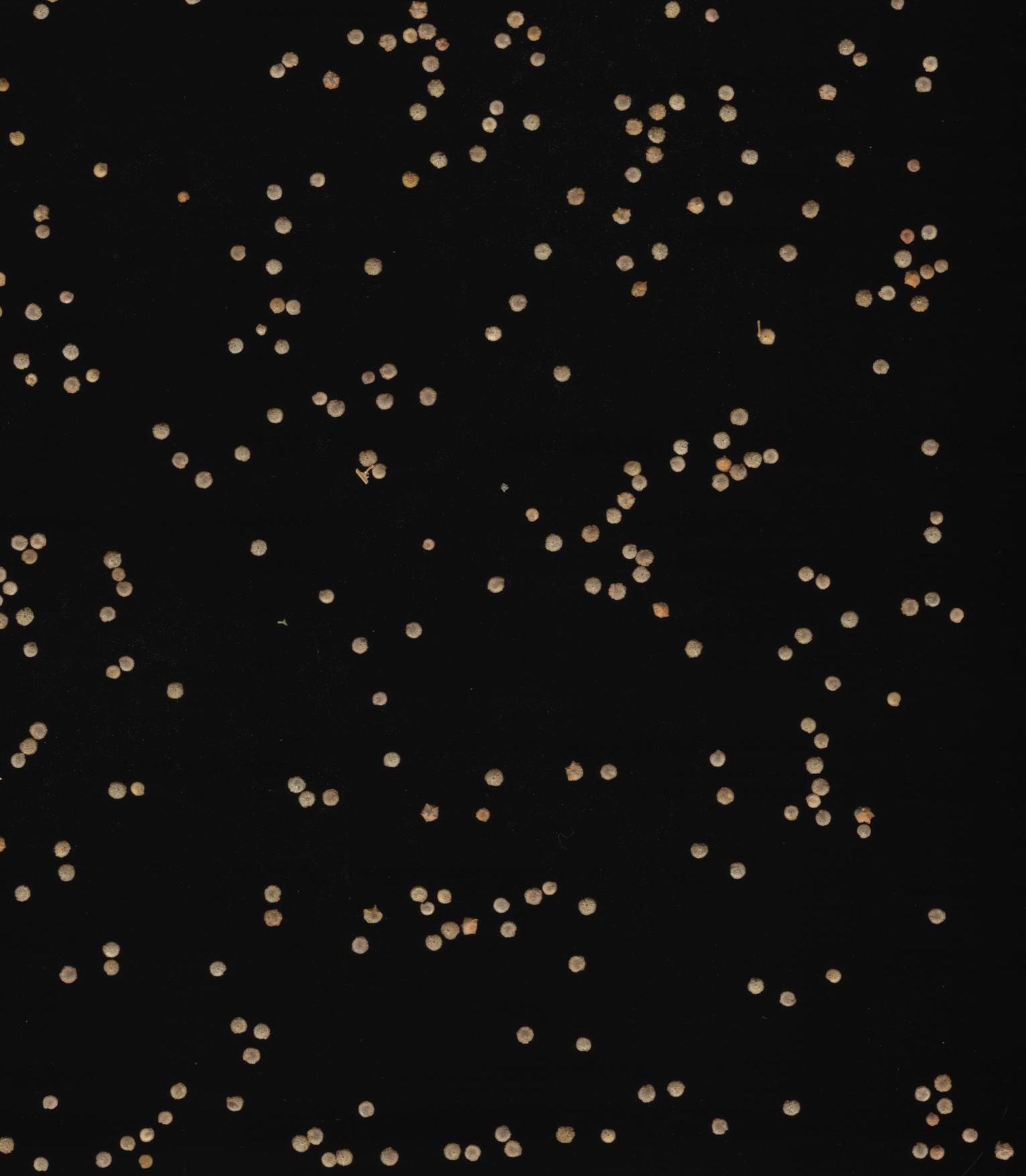

Supplement: Supplementary file 2 [file Presentation_2.zip › Presentation 2 updated/SM Figure S6E.JPEG]

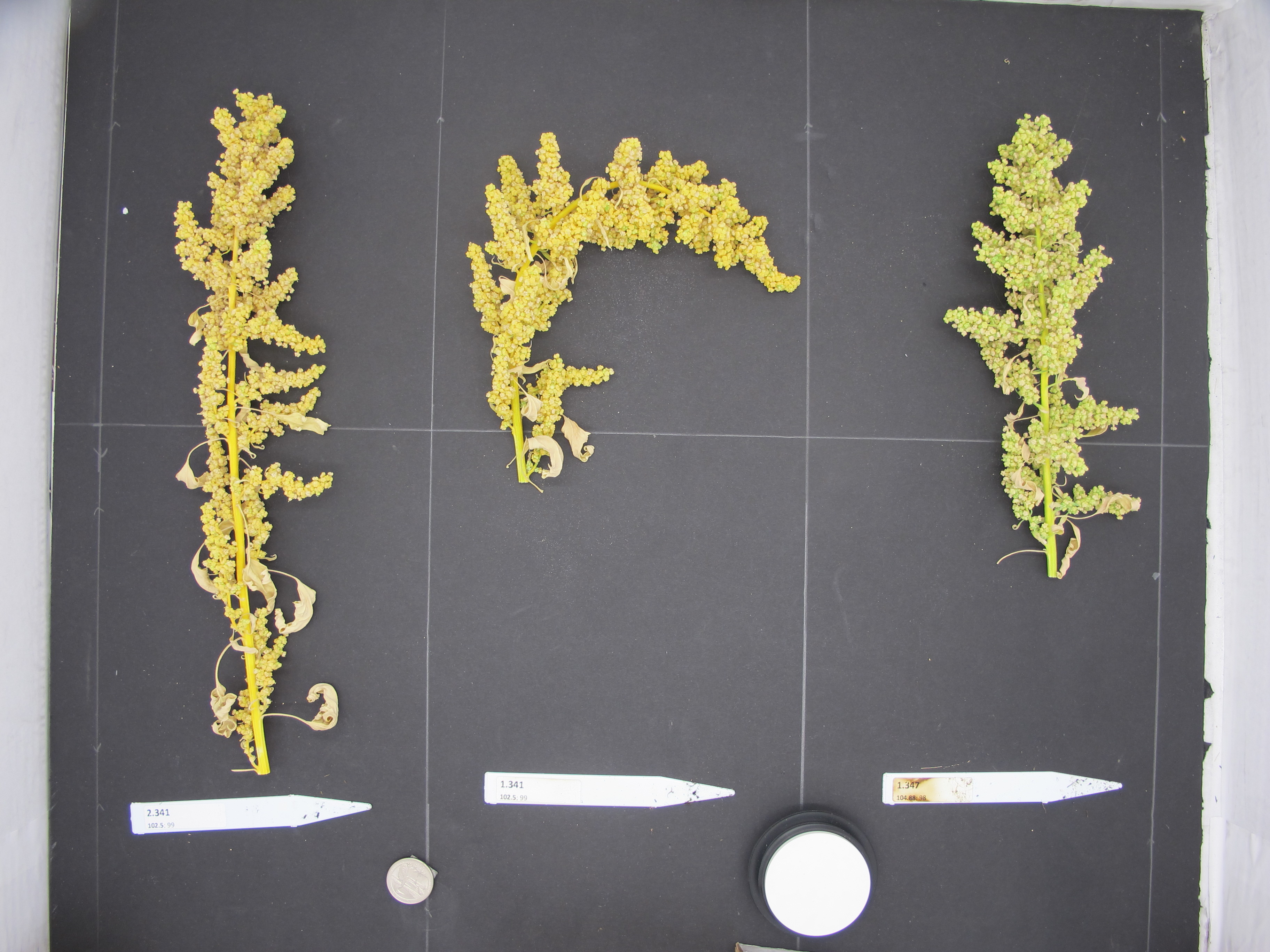

Supplement: Supplementary file 2 [file Presentation_2.zip › Presentation 2 updated/SM Figure S7.JPEG]

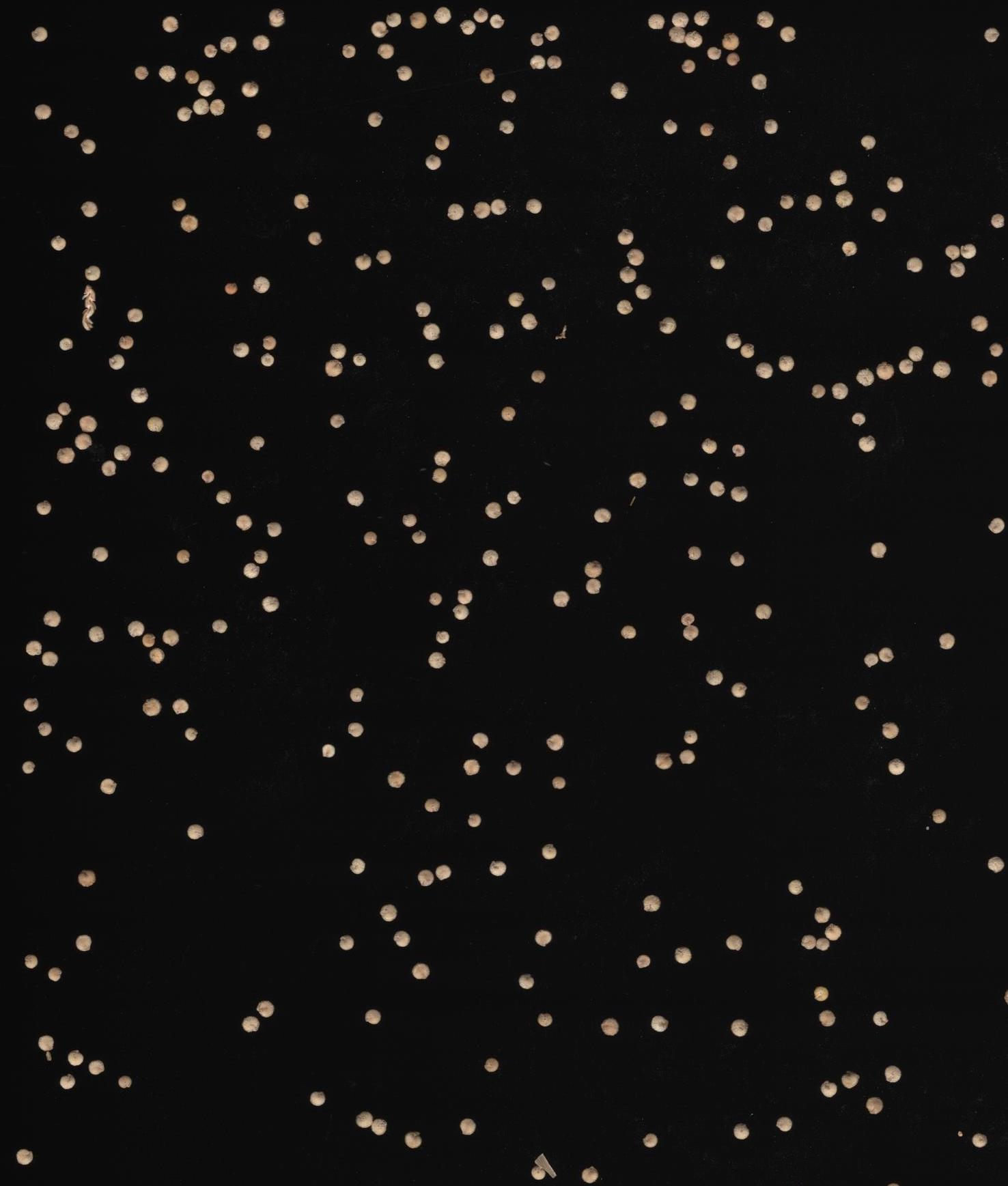

Supplement: Supplementary file 2 [file Presentation_2.zip › Presentation 2 updated/SM Figure S6D.JPEG]

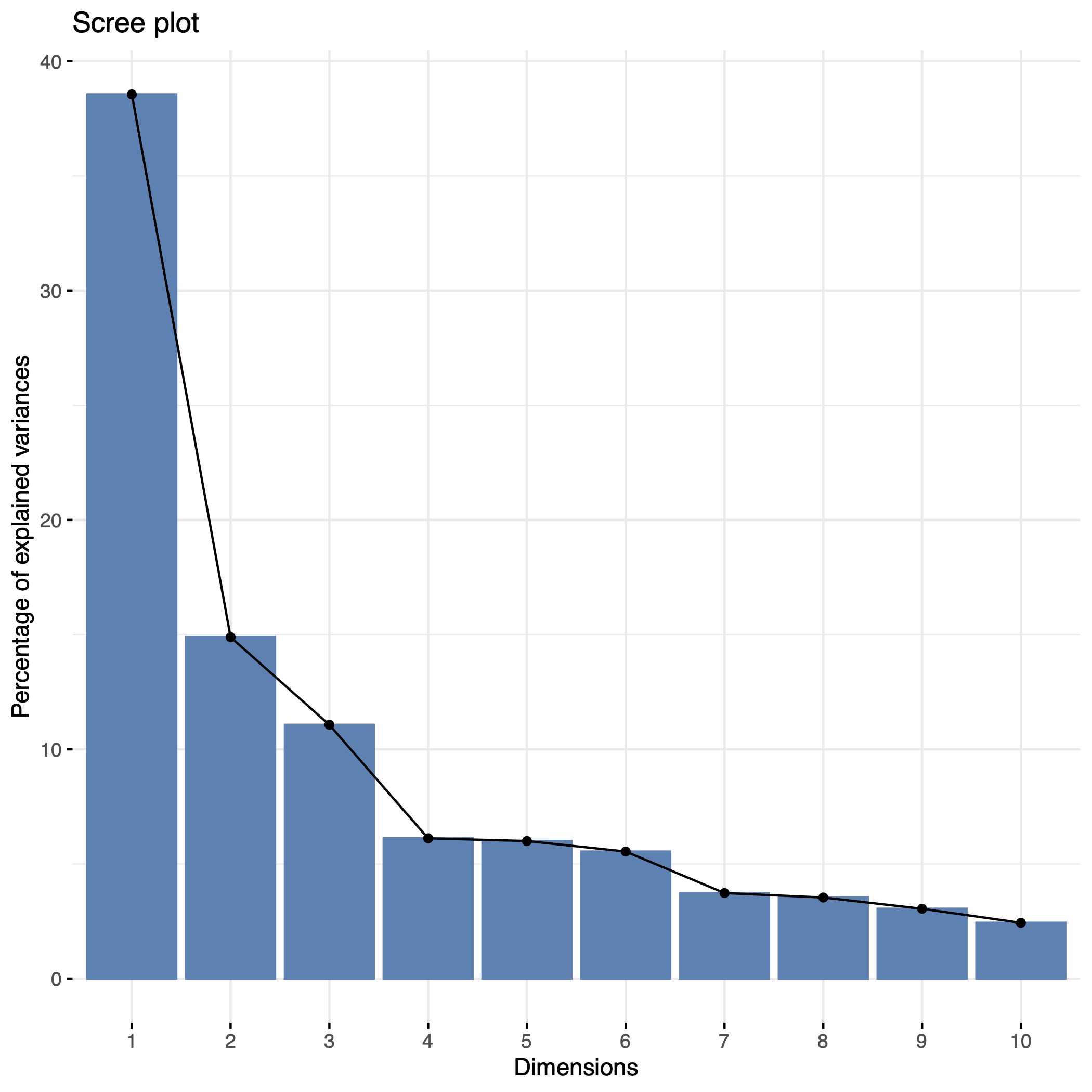

Supplement: Supplementary file 2 [file Presentation_2.zip › Presentation 2 updated/SM Figure S5.TIFF]

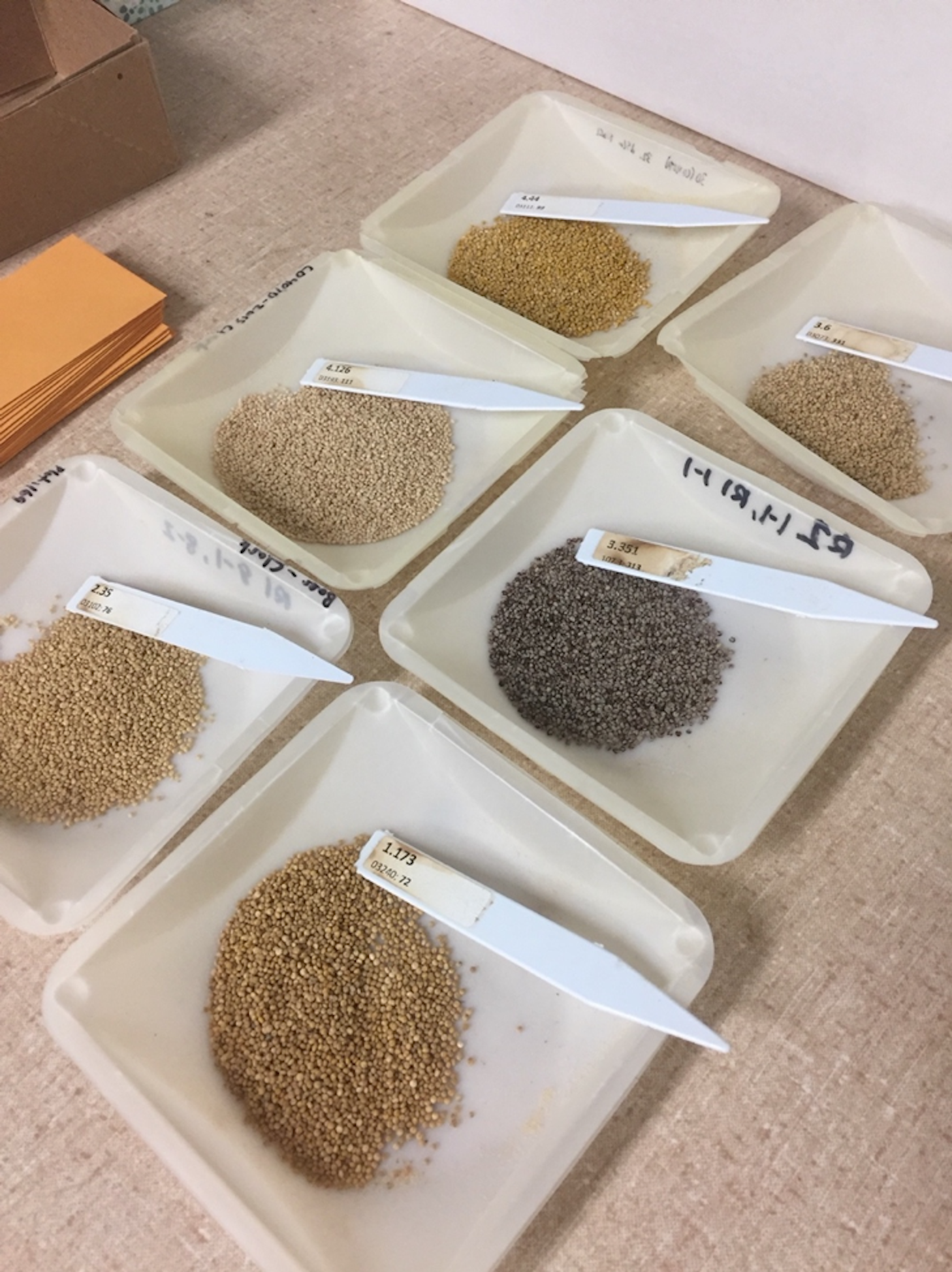

Supplement: Supplementary file 2 [file Presentation_2.zip › Presentation 2 updated/SM Figure S3.PNG]

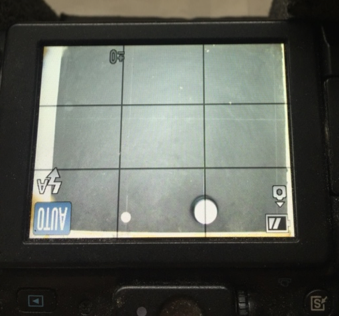

Supplement: Supplementary file 2 [file Presentation_2.zip › Presentation 2 updated/SM Figure S2A.PNG]

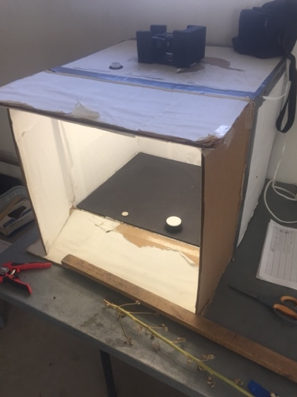

Supplement: Supplementary file 2 [file Presentation_2.zip › Presentation 2 updated/SM Figure S2B.PNG]

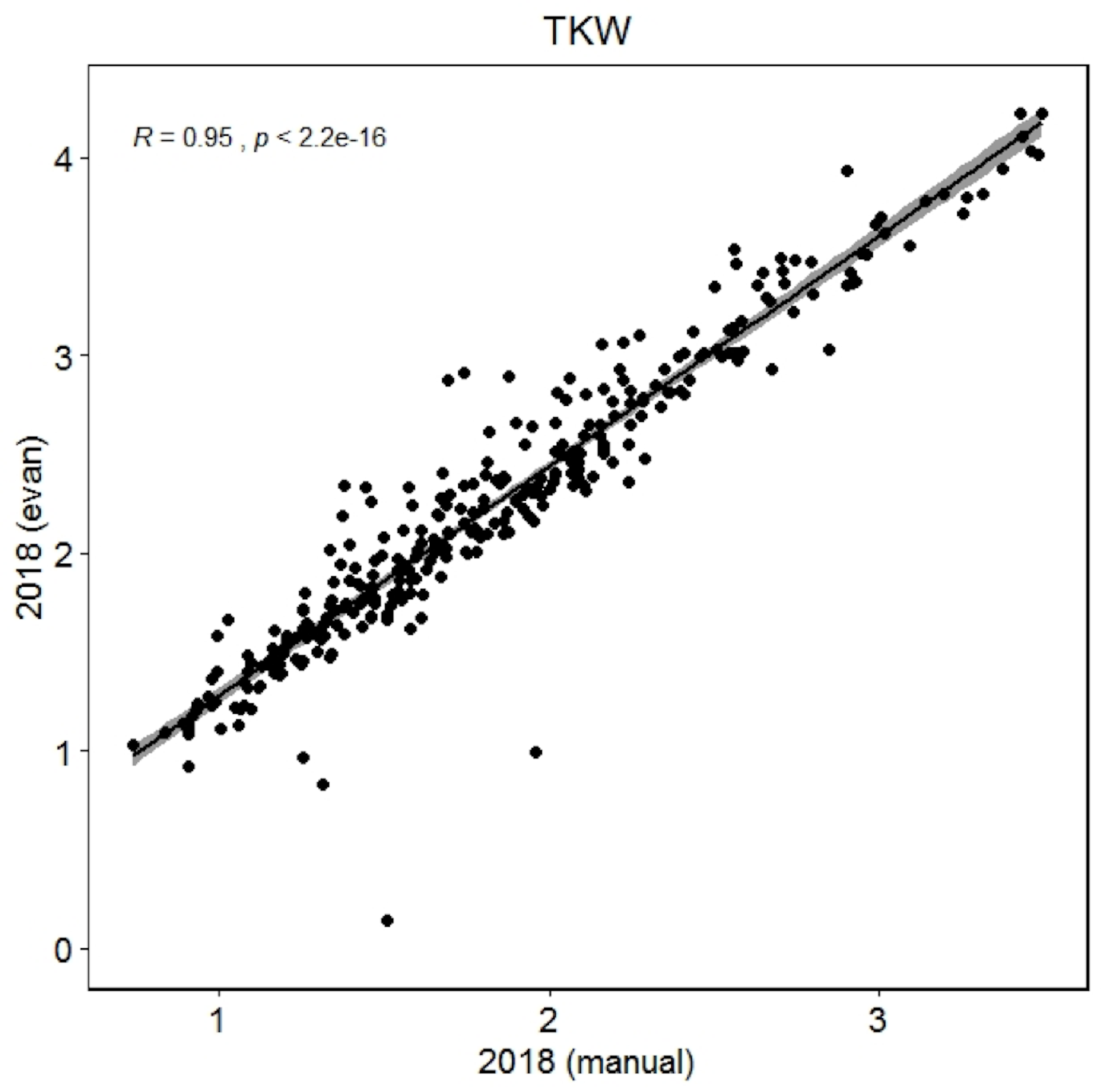

Supplement: Supplementary file 2 [file Presentation_2.zip › Presentation 2 updated/SM Figure S4.PNG]

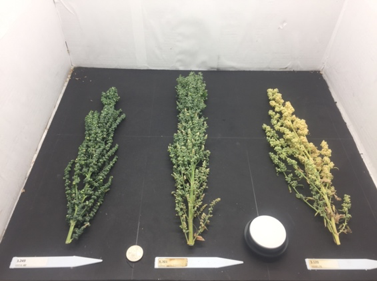

Supplement: Supplementary file 2 [file Presentation_2.zip › Presentation 2 updated/SM Figure S2C.PNG]

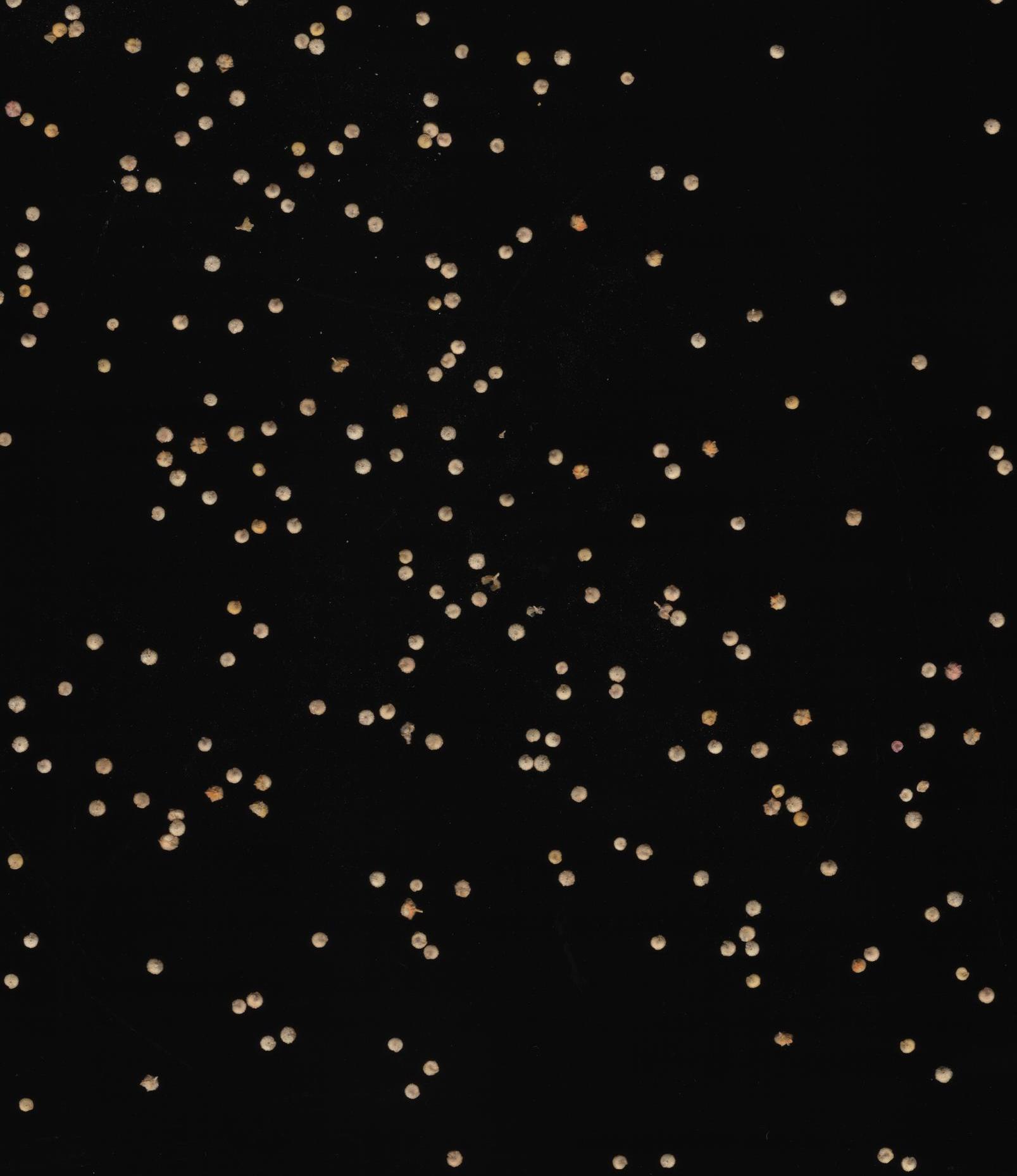

Supplement: Supplementary file 2 [file Presentation_2.zip › Presentation 2 updated/SM Figure S6F.JPEG]

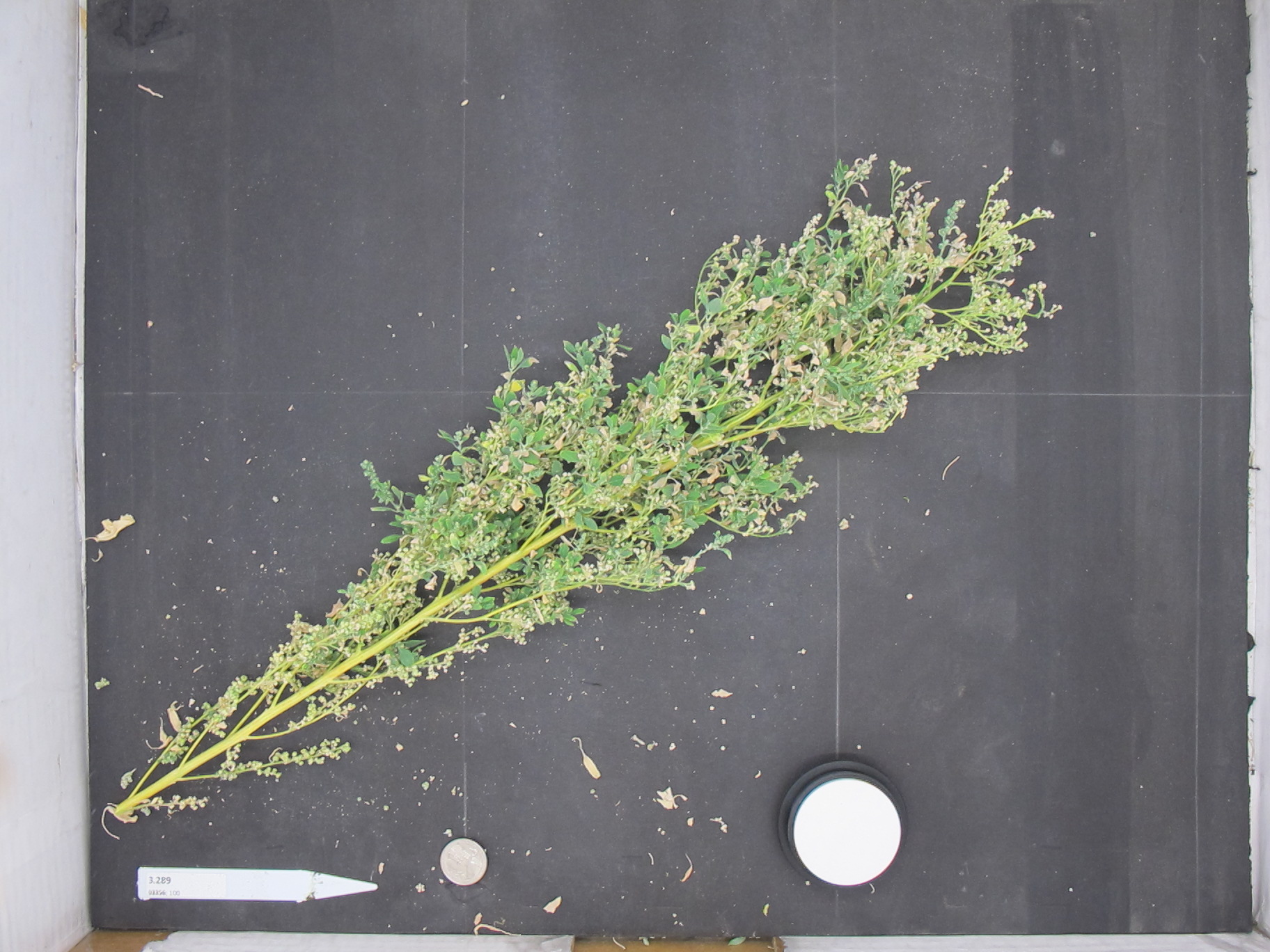

Supplement: Supplementary file 2 [file Presentation_2.zip › Presentation 2 updated/SM Figure S6A.JPEG]
