# Supplementary material for: A comprehensive characterization of agronomic and end-use quality phenotypes across a quinoa world core collection
Source: Front Plant Sci. 2023 Feb 16;14:1101547. doi: 10.3389/fpls.2023.1101547 (PMC9978749; doi:10.3389/fpls.2023.1101547)
Supplement: Supplementary file 3 [file DataSheet_1.docx]

Supplementary Material

# Supplementary Data

Supplementary data is provided in Frontiers_GH18_Supplementary_Material_rawData.csv and Frontiers_GH18_Supplementary_Material_greenhouseManagement.csv

# Supplementary Figures and Tables

## Supplementary Figures

**Supplementary Figure S1.** Phenotyping card illustrating scores for panicle color. Image credit: David Wu

**Supplementary Figure S2(A-B).** Panicle imaging setup. The light box was constructed from a cardboard box, two LED light strips (fluorescent linkable strip, 900 lumens, Lights of America, Chino, CA), and a matte black background (A). A camera (Canon PowerShot G12) was mounted to the top of the box (B). A size and reflectance standard were positioned below the panicles. Up to three panicles were imaged simultaneously, although some panicles were imaged individually (C). Photo credit: Evan Craine

**Supplementary Figure S3.** Seed samples following post-harvest seed cleaning process, before post-harvest phenotyping for seed composition and morphology traits. Image credit: Evan Craine

**Supplementary Figure S4.** Correlation between thousand seed weight (i.e. thousand kernel weight; TKW) as estimated using image analysis (i.e. 2018 [evan]), where the number of seeds is counted and the weight of those seeds is known, and hand-counted thousand seed weight (i.e. 2018 [manual]).

**Supplementary Figure S5.** Scree plot presenting the percentage of explained variances for dimensions 1 to 10.

## Supplementary Tables

**Supplementary Table S1.** Passport information for the quinoa accessions studied.

**Supplementary Table S2.** Greenhouse management information, including application of biological and chemnical controls and date of application.

**Supplementary Table S3.** Correlation matrix presenting Spearman correlation coefficients calculated using best linear unbiased estimates (BLUEs) are provided on the second sheet of the workbook, titled ‘TableS3_correlationMatrix’. Trait abbreviation on provided on the first sheet of the workbook, titled ‘metaData’.

**Supplementary Table S4.** Best linear unbiased estimates (BLUEs) provided for each accession, in addition to the collection and cluster identity according to all trait or three trait (yield, days to harvest, height at harvest). Rows are sorted by collection and then accession in alphabetical order. Trait abbreviations are provided on sheet one, titled ‘metaData’.

**Supplementary Table S5.** Amino acid scores (AAS) provided for each accession. AAS calculated as best linear unbiased estimates (BLUEs) of essential amino acids (abbreviated using standard three letter codes) divided by infant or adult daily requirements. The amino acid with the lowest score is listed (Limiting_AA), as well as additional limiting amino acids (Limiting_AAs), based on comparisons to both infant and adult requirements (FAO/WHO/UNU, 2007).
